# Design, Synthesis, and Bioevaluation of Moxifloxacin Hydrazide Metal Complexes: Integrated Spectroscopic, Computational, Antimicrobial, and Anticancer Investigations

**DOI:** 10.3390/ijms27073057

**Published:** 2026-03-27

**Authors:** Abbas Mamdoh Abbas, Sara Reda Fisal, Ibrahim A. I. Ali, W. Christopher Boyd, Haitham Kalil, Adel Sayed Orabi

**Affiliations:** 1 Department of Chemistry, Faculty of Science, Suez Canal University, Ismailia 41522, Egypt; sarareda@science.suez.edu.eg (S.R.F.); ibrahim3369@yahoo.com (I.A.I.A.); orabiadel@hotmail.com (A.S.O.); 2 Department of Chemistry, College of Arts and Sciences, Cleveland State University, Cleveland, OH 44115, USA; w.c.boyd59@csuohio.edu; 3 Department of Chemistry & Biochemistry, California Polytechnic State University, San Luis Obispo, CA 93407, USA

**Keywords:** moxifloxacin hydrazide, metal complexes, DFT, antimicrobial, anticancer

## Abstract

This study reports the synthesis, spectroscopic characterization, and biological evaluation of a novel moxifloxacin hydrazide derivative (MOX-H) and its metal complexes with Co(II), Ni(II), Cu(II), VO(IV), and Gd(III). The ligand was synthesized by hydrazinolysis of moxifloxacin hydrochloride, and the resulting hydrazide was subsequently complexed with the respective metal salts. The interaction between MOX-H and the metal ions yielded the corresponding complexes, formulated as [Co(H_2_O)Cl(MOX-H)_2_]Cl·2.5H_2_O, [Ni(H_2_O)Cl(MOX-H)_2_]Cl.4.5H_2_O, [VO(MOX-H)_2_]SO_4_.3.5H_2_O, [Gd (H_2_O)(MOX-H)_2_(NO_3_)_2_]NO_3_.2H_2_O, and [Cu(MOX-H)_2_(H_2_O)Cl]Cl·*x*H_2_O (where *x* = 2, 2.5, 0.5, for products synthesized via template, microwave-assisted, and hydrothermal methods, respectively). The synthesized analogues were characterized by elemental analysis (CHN), FT-IR, UV-visible, and ^1^H NMR spectroscopy, and mass spectrometry, as well as thermogravimetric (TG/DTG) and magnetic measurements. FT-IR spectra confirmed coordination through the hydrazide carbonyl and amine groups, while UV–visible and magnetic data indicated predominantly octahedral geometries. The thermal behavior exhibited multistep decomposition with activation parameters supporting exothermic processes. When compared to the free ligand, the metal complexes showed increased antimicrobial activity against both Gram-positive and Gram-negative bacteria and fungus species, particularly for the Co(II) and Cu(II) complexes, which showed the largest inhibition zones. The Cu(II)–MOX-H complex exhibited the lowest MIC values (4.88–9.76 µg/mL) among all tested compounds, confirming its outstanding antibacterial potency and high sensitivity compared to the free ligand and standard drug. Cytotoxicity assays demonstrated selective anticancer activity, with the Cu(II)–MOX-H complex showing the highest potency (IC_50_ ≈ 2.95 µM against MCF-7 and IC_50_ ≈ 0.98 µM against HepG-2), while maintaining minimal toxicity toward normal cells. These findings were corroborated by molecular docking investigations, which showed that the MOX-H complexes had substantial binding affinities (−9 to −10 kcal/mol) toward DNA topoisomerase II, consistent with their observed biological effects.

## 1. Introduction

One of the most important global health issues of the twenty-first century is antimicrobial resistance (AMR), demanding the development of novel and more potent antimicrobial agents. Among the available antibiotic classes, fluoroquinolones exert their action primarily by inhibiting bacterial DNA gyrase and topoisomerase IV, thus halting DNA replication and transcription. However, increasing antimicrobial resistance has prompted the search for structurally modified fluoroquinolones with enhanced antimicrobial efficacy and selectivity. One such candidate is moxifloxacin, a fourth-generation fluoroquinolone [1,2,3,4].

Among fluoroquinolones, moxifloxacin is promising owing to its structural features, including a fluorine atom at C-6, a methoxy group at C-8, and a diazabicyclo side chain at position 7, which collectively contribute to its potent and broad-spectrum activity against a variety of pathogens. The chemical modification of moxifloxacin has gained increasing attention as a strategy to overcome resistance, improve pharmacokinetic behavior, and expand its biological profile [5,6,7].

To enhance the therapeutic profile of moxifloxacin and address resistance, structural derivatization through hydrazinolysis, to yield moxifloxacin hydrazide (MOX-H), has proven to be an effective approach. This transformation preserves the quinolone core while introducing additional coordination sites, the carbonyl oxygen and the hydrazide nitrogen, enabling the formation of stable metal complexes. Metal complexation is known to strongly influence biological activity. Co(II), Ni(II), and Cu(II) were selected as representative first-row divalent ions, while VO(IV) was selected as a structurally diagnostic vanadyl center with a strongly defined axial V=O bond. Gd(III) was included as a trivalent, hard-acid comparison. These features can enhance cell permeability, target interaction (e.g., DNA binding), and overall antimicrobial or anticancer efficacy [7,8,9,10,11,12,13].

Numerous studies have shown that metal complexes derived from fluoroquinolone hydrazides exhibit superior antimicrobial and anticancer activities compared to their parent ligands. For instance, Cu(II) complexes have been reported to show strong DNA interaction and redox activity, which may contribute to cytotoxic effects; however, the precise mechanism remains dependent on the biological system affected. VO(IV) complexes have been reported to possess both antimicrobial and insulin-mimetic effects, while Gd(III)-based systems offer paramagnetic properties valuable in both therapy and diagnostics. These findings support the development of new MOX-H-based metal complexes to overcome the limitations of conventional moxifloxacin [4,12,13,14,15,16,17,18,19,20,21,22,23,24,25,26,27,28,29,30,31,32].

Although the coordination chemistry of fluoroquinolones has been extensively studied, systematic investigations which focus specifically on moxifloxacin hydrazide (MOX-H) as the ligand are virtually absent from literature. To the best of our knowledge, this study is the first comprehensive report employing moxifloxacin hydrazide (MOX-H) as a direct coordination scaffold to access a representative set of metal complexes spanning distinct coordination chemistry regimes. The complexes were characterized using complementary spectroscopic (FTIR, UV-visible, NMR, EPR), thermal, magnetic, and computational approaches. Beyond structural characterization, this work combines in vitro antimicrobial assays, cytotoxicity profiling, and molecular docking studies against topoisomerase II, offering a multi-dimensional biological evaluation.

This study introduces a new class of bioactive metal–quinolone hybrids with demonstrably enhanced antimicrobial and anticancer potential. The findings lay the groundwork for further development of MOX-H metal complexes as dual-function therapeutic candidates to address rising drug resistance.

## 2. Results and Discussion

### 2.1. Characterization of the Drug Derivative MOX-H

Thin-layer chromatography (TLC) with silica gel plates and a chloroform: ethanol (4:1 *v*/*v*) solvent solution was used to track the reaction’s purity and progress. The compound exhibited a single spot with an R_f_ value of 0.56, confirming the formation of a single product.

The product was obtained as a brownish-buff crystalline powder with a melting point of 222 °C and a yield of 76%. The molar mass was 415.47 g/mol, as confirmed by mass spectrometry, showing an [M]^+^ ion peak consistent with the proposed structure (Table 1, Figure S1).

The ligand showed good solubility in polar organic solvents (ethanol, methanol, DMSO, DMF), indicating its polar nature and the presence of hydrazide and carbonyl functional groups.

Elemental analysis (CHN) results were compared with the theoretical values for the proposed formula (C_21_H_24_FN_5_O_4_): C: calculated 60.71%; found 60.48%, H: calculated 6.31%; found 6.69%, and N: calculated 16.85%; found 16.42%. While the nitrogen percentage closely matches the theoretical value, the deviations in carbon and hydrogen suggest the possible presence of residual solvents, moisture, or partial hydration, especially since the sample was not subjected to extended vacuum drying. These factors may account for the underestimation of C and H content. Nevertheless, the discrepancies between the theoretical and experimental elemental analysis values are small overall, and the mass spectrometry data offer further support for the proposed molecular structure.

A significant molecular ion peak at *m*/*z* = 415 [M]^+^ was visible in the mass spectra of MOX-H (Figure S1), which was exactly in line with the compound’s estimated molar mass.

The clean fragmentation pattern and absence of unexpected peaks strongly support the structural integrity and purity of the synthesized ligand.

**Figure 1:**
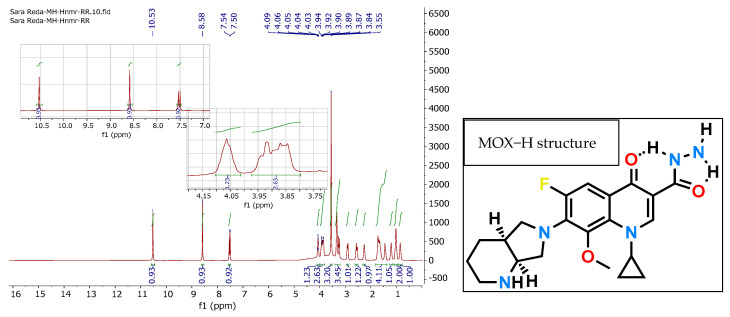
The ^1^H NMR spectrum of the free MOX−H ligand.

The ^1^H NMR spectrum of MOX-H (400 MHz, DMSO-*d*_6_, TMS; Figure 1) is fully consistent with the proposed hydrazide structure and integrates to 26H: δ 10.53 (s, 1H, hydrazide CONH, D_2_O-exchangeable), 8.58 (s, 1H, quinolone Ar–H), 7.52 (d, 1H, quinolone Ar–H, influenced by adjacent F/C=O), 4.03 (m, 1H, cyclopropyl CH), 3.94–3.84 (br m, 5H, overlapping –NH_2_ of the hydrazide, 2H, D_2_O-exchangeable, with the 8-OCH_3_, 3H), 3.10–2.90 (br, 1H, ring NH of the diazabicyclic moiety, D_2_O-exchangeable), 3.60–2.60 (m, 12H, diazabicyclic NCH/NCH_2_), 1.30–0.90 (m, 4H, cyclopropyl 2 × CH_2_). The residual DMSO-*d*_6_ peak appears at δ 2.50 (not integrated). The broadness of the NH/NH_2_ signals and the downfield CONH (δ 10.53) reflect hydrogen bonding and partial exchange in DMSO-*d*_6_. A brief D_2_O shake decreases the total integral by 4H (1 × CONH + 2 × NH_2_ + 1 × ring NH), leaving a clean OCH_3_ (3H) within the 3.94–3.84 ppm envelope. Overall, the chemical shifts, multiplicities, and integrations confirm the successful formation of the MOX-H hydrazide derivative [14,15].

Several key changes in the FT-IR spectrum supported the successful chemical modification. The broad absorption envelope observed in the 3400–2900 cm^−1^ region was assigned to overlapping O–H and N–H stretching vibrations, broadened by hydrogen bonding and possible associated water molecules, together with contributions from aliphatic or aromatic C–H stretching modes in the lower part of this range. A notable shift to lower wavenumber (red shift) in the amide C=O stretching vibration was observed, moving from 1708 cm^−1^ in the parent MOX-HCl to 1665 cm^−1^ in MOX-H, indicating amide bond formation. Furthermore, the appearance of a new absorption band at 1060 cm^−1^, characteristic of N–N stretching, confirmed the introduction of the hydrazide moiety. The presence of a C=N stretching vibration at 1587 cm^−1^ also suggested resonance delocalization of the π-bond system, potentially involving keto–enol tautomerism within the hydrazide framework [15,16,17]. Importantly, the C=O stretch associated with the quinolone ring remained largely unaffected, confirming that the core scaffold of the moxifloxacin molecule was retained throughout the reaction process.

These IR findings were fully supported by ^1^H NMR spectroscopy, which showed the appearance of new, high-wavenumber signals corresponding to NH and NH_2_ protons, and which were consistent with the proposed substitution pattern. Additionally, MM2 molecular mechanics simulations revealed a more stable, low-energy conformation for the hydrazide, further stabilized by intramolecular hydrogen bonding interactions, as shown in NMR of MOX-H.

Collectively, the combined spectroscopic, theoretical, and structural data confirm the successful conversion of MOX-HCl to MOX-H. A detailed comparison of IR bands before and after complexation is presented in Table 2, while further insights into metal coordination behavior of the ligand are illustrated in Figures S2 and S3.

The produced moxifloxacin derivatives’ powder X-ray diffraction (PXRD) patterns (Figure S4 and Table S1) showed notable differences in crystallinity and structural organization throughout the series. Among the compounds examined, the MOX-H ligand demonstrated the highest degree of crystallinity, evidenced by multiple sharp and intense diffraction peaks located at 2θ = 7.5°, 11.0°, 17.9°, 22.1°, 26.5°, 28.2°, and 31.3°. These reflections indicate a high degree of long-range molecular order and a well-organized, periodic crystal lattice. The sharpness and intensity of the diffraction peaks confirm the presence of a single, well-crystallized phase, likely corresponding to a monoclinic or triclinic crystal system, as suggested by preliminary indexing results (M20 = 14, unit cell volume = 527.69 Å^3^). Additional crystallographic parameters extracted from the PXRD analysis further support this conclusion. MOX-H exhibited an average crystallite size of 11.53 nm, with microstrain (δ) values ranging from 1.59 × 10^−2^ to 8.65 × 10^−2^, indicating low structural distortion within the lattice. The calculated number of unit cells (Z) was 1, and the compound was classified as monoclinic, consistent with the indexing results. These findings collectively confirm that MOX-H is highly crystalline, showing a well-defined and stable crystalline architecture compared to the other derivatives in the series.

Further structural refinement of the XRD data was performed using iterative cycles in Expo2014, yielding accurate Miller indices, *d*-spacing values, and unit cell parameters (Figures S5 and S6 and Table S2). These results provide compelling evidence for the formation of a well-defined crystalline phase, supporting the assignment of a monoclinic crystal structure for the MOX-H ligand [18].

### 2.2. Characterization of Metal Complexes

#### 2.2.1. Physicochemical Properties

Coordination of moxifloxacin hydrazide (MOX-H) with a range of metal ions, namely, Co(II), Ni(II), Cu(II), VO(IV), and Gd(III), resulted in the formation of stable crystalline complexes with the following general formulas: Co(H_2_O)Cl(MOX-H)_2_]Cl·2.5H_2_O, [Ni(H_2_O)Cl(MOX-H)_2_]Cl·4.5H_2_O, [VO(MOX-H)_2_]SO_4_·3.5H_2_O, [Gd(H_2_O)(MOX-H)_2_(NO_3_)_2_]NO_3_·2H_2_O, and [Cu(MOX-H)_2_(H_2_O)Cl]Cl·*x*H_2_O (where *x* = 2, 2.5, 0.5, for products synthesized via template, microwave-assisted, and hydrothermal methods, respectively).

Because single crystals suitable for X-ray diffraction could not be obtained, the structures discussed herein are proposed formulations, rather than structures confirmed by crystallography. However, these proposed structures are strongly supported by combined metal analysis, molar conductivity, FT-IR, UV–visible, EPR, magnetic, and thermal data.

All complexes appeared as crystalline solids with distinct characteristic colors: brick brown for Co(II), dark green for Ni(II), reddish brown for VO(IV), and greenish yellow for Gd(III), as presented in Table 1. The Cu(II) complexes exhibited dirty green coloration, with minor variations in intensity depending on the synthetic method.

While the free ligand had a melting point of 220 °C, the metal complexes displayed diverse melting points reflecting the intermolecular interactions in their solid-state structures. Complexes with melting points greater than 280 °C (e.g., Co and Ni complexes) suggest strong metal–ligand interactions and enhanced lattice rigidity, whereas those melting between 130–212 °C exhibit weaker intermolecular forces, possibly due to coordinated solvent molecules. Notably, the Gd(III) complex, with a melting point of 218 °C, is similar to the free ligand, indicating a limited structural influence from coordination. This result is in keeping with the general view according to which the 4*f* electrons are not significantly involved in covalent bonding in lanthanide complexes [19].

All synthesized complexes were soluble in water and DMSO, facilitating further analytical and biological studies. Molar conductivity measurements in 10^−3^ M solutions in DMSO revealed values between 20 and 97 Ω^−1^·cm^2^·mol^−1^, reflecting diverse electrolytic behaviors. Co(II), Ni(II), and Cu(II) complexes showed moderate conductivities (40–53 Ω^−1^·cm^2^·mol^−1^), consistent with a 1:1 electrolytic nature, suggesting the presence of one dissociable chloride ion in the outer coordination sphere. The Gd(III) complex exhibited the highest conductivity (97 Ω^−1^·cm^2^·mol^−1^), characteristic of a 1:1 electrolyte, consistent with the presence of one nitrate ions in the outer coordination sphere [20,21].

These results are in excellent agreement with the proposed molecular formulas and the suggested coordination environments for the metal centers. Furthermore, metal content analysis (M%) by complexometric titration and thermogravimetric methods demonstrated close agreement with theoretical values, reinforcing the proposed stoichiometries of the complexes.

#### 2.2.2. FT-IR Spectra

The infrared spectra of the synthesized metal complexes of Co(II), Ni(II), Cu(II), VO(IV), and Gd(III) exhibited notable deviations from that of the free ligand MOX-H, confirming successful complex formation through specific donor sites. In the spectrum of MOX-H, a broad absorption band centered around 3430 and 3238 cm^−1^ corresponds to the O–H and N–H (hydrazide/NH_2_) stretching vibrations. These bands were retained in the spectra of all metal complexes with minor shifts, suggesting the presence of both coordinated and lattice water molecules, and indicating that the N–H groups are involved in coordination with the metal centers. The amide carbonyl stretching vibration (C=O), initially observed at 1665 cm^−1^ in MOX-H, showed significant weakening or complete disappearance in the complexes. This suggests the involvement of the amide oxygen atom in chelation, a conclusion further supported by the shift in the adjacent C=N/azomethine region. The C=O stretch of the quinolone ring, detected at 1621 cm^−1^ in MOX-H, persisted in the complexes with varying intensities, indicating limited participation of this group in coordination. This observation implies that chelation predominantly involves the hydrazide moiety rather than the aromatic carbonyl [17,22,23].

**Figure 2:**
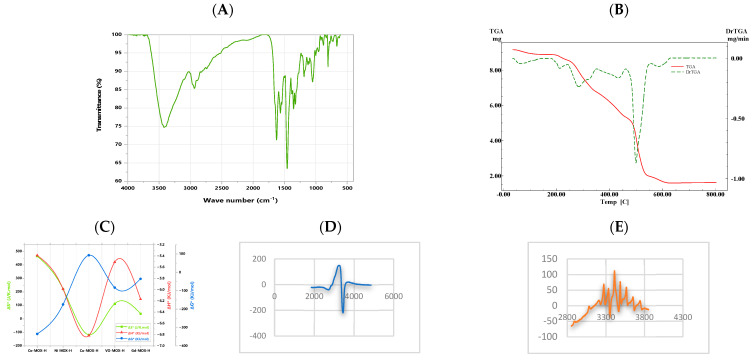
(**A**) FT-IR spectrum of [Ni(MOX-H)_2_(H_2_O)Cl]Cl·4.5H_2_O; (**B**) TGA and DTG curves of [Gd(MOX−H)_2_(H_2_O)(NO_3_)_2_]NO_3_.2H_2_O; (**C**) Variation of Δ*G*‡ and Δ*S*‡ parameters accompanied by the liberation of coordinated water; (**D**) EPR spectrum for Cu(II)−MOX−H complex; (**E**) EPR spectrum for VO(II)−MOX−H complex.

The FT-IR spectrum of the Gd(III) complex exhibits a strong nitrate ν_3_ band at 1384 cm^−1^, together with bands at 1050 cm^−1^ (ν_1_) and 770 cm^−1^ (assigned to ν_4_). The large separation Δν = ν_3_ − ν_4_ = 614 cm^−1^, combined with the high molar conductivity (Λₘ = 97 Ω^−1^·cm^2^·mol^−1^, 1:1 electrolyte), indicates the presence of mixed nitrate environments, predominantly outer-sphere (ionic) nitrate, alongside a coordinated, monodentate nitrate ligand [24]. The VO(IV) complex displayed a characteristic V=O stretching band at 827 cm^−1^, affirming the presence of the oxovanadium (IV) moiety. The free sulfate ion belongs to the highly symmetrical point group *T_d_*, and of its four vibrational frequencies, only ν_3_ and ν_4_ are active in the IR. Additionally, weak bands in the 600–500 cm^−1^ region were attributed to M–O and M–N stretching vibrations, confirming coordination through oxygen and nitrogen donor atoms [25,26,27]. These spectral changes collectively highlight the shift in electron density upon complexation and provide insight into the mode of coordination. The spectral data are summarized in Table 2, with an example of Ni-MOX complex spectra shown in Figure 2A and other complexes are presented in Figures S7–S13.

#### 2.2.3. Thermal Analysis

We examined MOX-H and its metal complexes by TGA/DTG under N_2_ flow (Figure 2B and Figures S14–S20; Table S3). In short, the ligand thermolysis occurs in three stages, and each complex follows a three-step pattern that mirrors its hydration and counter-ions.

The first step occurs between 31–364 °C (DTG = 278 °C) and corresponds to mass loss of 17.4% (calcd. 17.4%), attributable to initial decomposition of the ligand. The second step, in the range 364–465 °C (DTG = 432 °C), shows a further mass loss of 15.4% (calcd. 15.4%) associated with the onset of degradation of the hydrazide framework. In the final step, from 465–650 °C (DTG = 559 °C), thermolysis of the remaining organic moiety is completed, and no residual mass is detected at approximately 650 °C.

All MOX-H metal complexes exhibit a similar thermal decomposition pattern, involving initial loss of lattice water, followed by elimination of coordinated species concurrent with partial degradation of the organic framework, and finally conversion to simple inorganic residues. In the first stage, the experimental mass losses are in good agreement with those calculated for the proposed hydrated formulations. Dehydration commences at approximately 64 °C for the Co(II) complex, 71 °C for the Ni(II) complex, around 56/68/68 °C for the three Cu(II) preparations (a/b/c), 72 °C for the VO(IV) complex, and 62 °C for the Gd(III) complex. The observed versus calculated mass losses are 4.48/4.39% for Co(II), 7.83/7.65% for Ni(II), 3.80/3.50% for Cu(II)(a), 4.57/4.37% for Cu(II)(b), 1.06/0.906% for Cu(II)(c), 6.17/5.96% for VO(IV), and 2.87/3.08% for Gd(III), which are consistent with the presence of 2.5, 4.5, 2.0/2.5/0.5, 3.5, and 2.0 molecules of lattice water, respectively.

The second stage is broader and carries the fingerprint of each coordination environment. Co(II) shows a single DTG maximum at 299 °C, while Ni(II) splits into two at 302 and 422 °C. The three Cu(II) samples give route-dependent envelopes: 283/338/446 °C for Cu(a), 176/304/347 °C for Cu(b), and 273/306 °C for Cu(c). VO(IV) shows two features at 260 and 315 °C, and Gd(III) gives a triplet at 213, 283, and 432 °C.

Interpreting these bands against the formulas is straightforward at the level of stoichiometric mass balance from the TG/DTG steps, noting that TG/DTG tracks mass loss but does not unambiguously identify evolved gases without hyphenated analysis (e.g., TG–FTIR or TG–MS). Co(II) and Ni(II) each release roughly one coordinated water molecule along with chloride-associated volatilization (commonly reported as HCl [28,29]), although the specific chlorine-containing gas cannot be confirmed from TG alone, while the Cu(II) complexes behave similarly (coordinated H_2_O and chloride-associated volatilization), with the extra peaks according to preparation methods. For VO(IV), the inner-sphere sulfate is expected to undergo sulfate-associated thermal decomposition that may generate SOx under sufficiently forcing conditions; however, the specific SO_2_ vs. SO_3_ assignment and the exact redox pathway cannot be established from TG/DTG alone, overlapping with the early stages of ligand breakdown. In Gd(III), the mid-temperature region carries the loss of one coordinated water and one coordinated nitrate, fully in line with the mixed-nitrate model supported by IR and conductivity.

By the final stage, the remaining organic material is consumed and whatever anion is left is expelled, leaving simple residues that make chemical sense. Co(II) ends as CoO after an onset near 406 °C; Ni(II) gives NiO with carbon at 451 °C; the Cu(II) series finishes as CuO for Cu(a) (462 °C) and Cu(c) (436 °C), but as metallic Cu for Cu(b) (445 °C), hinting at partial reduction under the experimental conditions. VO(IV) yields V_2_O_3_ with carbon from 411 °C, and the Gd(III) complex releases its two outer-sphere nitrates from 462 °C as thermolysis completes to a Gd and carbon residue.

The high-temperature residue plateau in TGA reflects the non-volatile inorganic fraction of the sample. Metal content from TG was estimated by mass balance from the residue percentage relative to the initial complex mass, using the common assumption that the residue at the plateau is dominated by a metal-oxide/oxo-inorganic phase and applying the corresponding stoichiometric relation between residue mass and metal mass [29]. This is a standard quantitative use of TGA residue in materials/coordination solids. Because TGA alone does not establish the crystalline identity of the residue, these TG-derived metal percentages are presented as model-based estimates; definitive residue identification would require residue PXRD and/or coupled evolved-gas/residue analysis, which are outside the scope of this work.

Kinetic and thermodynamic parameters.

Thermogravimetric data of MOX-H and its metal complexes were evaluated using the Coats–Redfern method, and the derived kinetic parameters—activation energy (*E*ₐ), entropy (Δ*S^‡^*), enthalpy (Δ*H^‡^*), Gibbs free energy (Δ*G^‡^*), and frequency factor (*Z*)—provided valuable insights into their thermal stability (Table S4 and Figure S21).

The decomposition stages were selected based on the most prominent mass-loss steps for each complex. In general, the Co(II), Ni(II), and VO(II) complexes exhibited higher activation energies, reflecting their greater stability and higher resistance to thermal degradation compared with the parent ligand. Conversely, MOX-H itself showed the lowest *E*_a_ and a less regular decomposition pattern, indicating a more labile structure and reduced stability. Negative values of Δ*S^‡^* observed for the ligand and most complexes suggest a decrease in randomness in the transition state, implying a more ordered structure during decomposition. The VO(II) complex displayed the most negative Δ*S^‡^*, denoting a highly ordered transition configuration, while the Cu(II) complex also exhibited a negative Δ*S^‡^* coupled with high Δ*G*^‡^, confirming its non-spontaneous and thermally stable behavior. The enthalpy changes (*ΔH*^‡^) were negative for all compounds, revealing an exothermic decomposition process. The Cu(II) complex, with Δ*H*^‡^ = –8.52 kJ mol^−1^, showed the highest energy release, reflecting a more favorable thermodynamic pathway. Variations in the pre-exponential factor (*Z*) indicated differences in collision frequency and molecular orientation during decomposition; higher *Z* values for Co–MOXH suggested a greater probability of decomposition per collision event [30]. Overall, the negative Δ*G*^‡^ values across most systems confirmed that the decomposition processes were thermodynamically feasible but non-spontaneous, requiring energy input to proceed. The Cu(II) complex had the largest Δ*G*^‡^ value of all, indicating that it was more stable than the other complexes. Figure 2C shows the variation in the Δ*G*^‡^ and Δ*S*^‡^ values based on the release of coordinated water.

#### 2.2.4. Magnetic Moment, UV-Visible Spectroscopy, and EPR Analysis

Because reliable EPR under our measurement conditions is most diagnostic for S = 1/2 centers, EPR was acquired for the Cu(II) and VO(IV) complexes only (Figure 2D,E and Table S5).

The Cu(II) spectrum is the classic axial case: $g_{∥}=2.263$ and $g_{⊥}=2.026$ ($g_{av}=2.105$) with a large $A_{∥}=190\times 10^{-4}\;{\mathrm{cm}}^{-1}$. The EPR ordering $g_{∥}>g_{⊥}>2.0023\;$ is consistent with a tetragonally elongated (Jahn–Teller–distorted) Cu(II) center in which the ground electronic state contains an unpaired electron in the $d_{x^{2}-y^{2}}$ orbital; this corresponds to an elongated-octahedral environment with strong equatorial O/N donors from MOX-H and a comparatively weaker axial interaction. The Kivelson exchange parameter is high (G=11.0), which tells us that exchange coupling is negligible, and that local ligand-field anisotropy is observed. Two bonding metrics back this picture: the orbital-reduction factor $K_{∥}=0.605$ ($E_{d-d}=184\;{\mathrm{kJ}\;\mathrm{mol}}^{-1}$) shows appreciable spin–orbit reduction by covalent mixing, and the Kivelson–Neiman σ-covalency parameter $\alpha^{2}=0.84$ points to moderate in-plane covalency typical for O/N-donor equatorial sets [31,32,33].

The Cu(II) spectrum is an axial envelope; the Cu hyperfine structure is not fully resolved under the present solid-state EPR conditions. Such partial/absent hyperfine resolution can occur due to linewidth broadening contributions (including g-strain and dipolar/inhomogeneous broadening) in powder/solid spectra, even for mononuclear Cu(II) sites [34]. The fitted parameters ($g_{∥}=2.263$, $g_{⊥}=2.026$, $g_{\mathrm{av}}=2.105$, $A_{∥}=190\times 10^{-4}\;{\mathrm{cm}}^{-1}$) are consistent with an axially elongated Cu(II) site described by the conventional g/A tensor framework. The exchange diagnostic $G=(g_{∥}-2)/(g_{⊥}-2)$ gives G≈10.12, which is well above 4 and therefore does not indicate significant Cu–Cu exchange coupling by the Procter–Hathaway criterion [35]. Taken together with the single-Cu analytical formulation and 1:1 electrolytic behavior, these data are most consistent with a predominantly mononuclear Cu(II) formulation under the conditions studied; however, we do not claim that solid-state CW-EPR alone provides definitive proof of nuclearity.

The EPR spectrum of the oxovanadium(IV) complex shows the characteristic vanadyl axial pattern, with $g_{∥}=1.96$ and $g_{⊥}=1.98$, together with hyperfine coupling constants of $A_{∥}=167\times 10^{-4}\;{\mathrm{cm}}^{-1}$ and $A_{⊥}=60.6\times 10^{-4}\;{\mathrm{cm}}^{-1}$. The relationships $g_{∥}<g_{⊥}$ and $A_{∥}>A_{⊥}$ are diagnostic of a $d_{xy}$ ground state that contains an unpaired electron, confirming a vanadyl center in which the strong V=O bond defines the principal axis and O/N donors occupy the equatorial plane, consistent with a square-pyramidal geometry. The average g-value ($g_{\mathrm{av}}=1.97$) lies well below the free-electron value (2.0023), reflecting significant ligand-field effects and metal–ligand orbital mixing. The covalency factor $k=A_{\mathrm{iso}}/P_{0}=0.92$ ($P_{0}{(}^{51}V)=0.0104\;{\mathrm{cm}}^{-1}$) indicates appreciable, though moderate, equatorial covalency alongside a robust V=O bond, in agreement with the ν(V=O) IR band and the LMCT transition near 355 nm. The exchange interaction parameter (*G* = 1.90) further suggests non-negligible magnetic exchange between neighboring vanadium centers in the solid state [36,37,38]. Accordingly, weak solid-state associations or intermolecular magnetic interaction cannot be completely excluded. However, we cautiously interpret the EPR data as supporting a vanadyl center in a square-pyramidal environment, rather than as definitive proof of mononuclearity.

The room-temperature magnetic moment and UV–visible spectral data of the synthesized MOX-H metal complexes are summarized in Table S6 and illustrated in Figures S22–S31. The Co(II) and Ni(II) derivatives behave like classic high-spin octahedral systems: each shows a set of weak, low-energy *d*–*d* transitions in the visible/near-IR and much stronger near-UV bands that we assign to O/N→M charge transfer. Their effective magnetic moments fall where predicted for *S* = 3/2 (Co) and *S* = 1 (Ni), neatly supporting the octahedral formulations with inner-sphere O/N coordination and an outer-sphere chloride counter-ion inferred from conductivity.

The Cu(II) complex spectrum collapses to a single broad *d*–*d* envelope in the red/near-IR, the hallmark of a tetragonally split $d_{x^{2}-y^{2}}\;$ ground state with a single unpaired electron, superimposed on intense LMCT bands in the near-UV. EPR measurements support this conclusion with an axial set $\left(g_{∥}>g_{⊥}>2.0023\right)$ and a substantial $A_{∥}$; the derived parameters (large G, $\alpha^{2}\approx 0.84$, $K_{∥}\approx 0.61$) point to a Jahn–Teller-elongated Cu(II) center—elongated-octahedral—with moderately covalent O/N equatorial bonds.

For VO(IV), the spectrum is dominated by a strong band at 355 nm, which is too energetic for a *d*–*d* transition and is therefore assigned to LMCT associated with the vanadyl unit. Any *d*–*d* feature is correspondingly weak and shifted to lower energy. The axial ESR pattern $\left(g_{∥}<g_{⊥},\;A_{∥}>A_{⊥}\right)$ and the covalency factor k≈0.92 confirm what is hinted at by the IR spectrum, namely, a robust V=O bond defining the *z*-axis and an O/N equatorial plane, i.e., a vanadyl square-pyramidal field consistent with inner-sphere sulfate.

Finally, Gd(III) shows the feature expected of a 4*f*^7^ metal center: weak, largely featureless *f*–*f* absorption and a spin-only magnetic moment close to the theoretical value, signaling an essentially ionic center with oxygen donors and minimal ligand-field perturbation [39].

The UV–visible spectra and magnetic data align cleanly with the IR/EPR/conductivity evidence: Co(II) and Ni(II) are high-spin octahedral; Cu(II) is axially elongated with a $d_{x^{2}-y^{2}}$ ground state [40]; VO(IV) is a vanadyl center with a strong V=O axis and LMCT in the near-UV; and Gd(III) shown spin-only magnetic properties. The convergence of these independent probes gives high confidence in the coordination structures assigned across the series.

**Figure 3:**
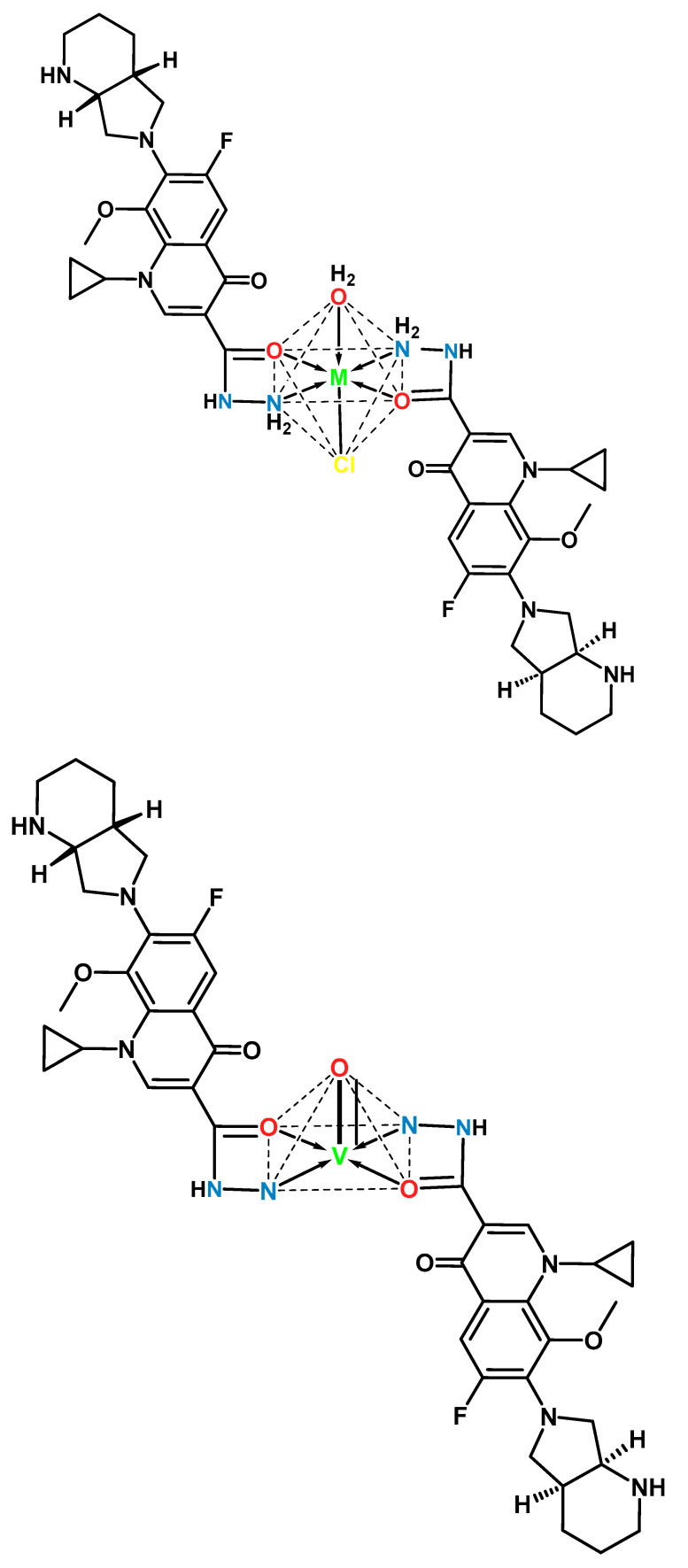

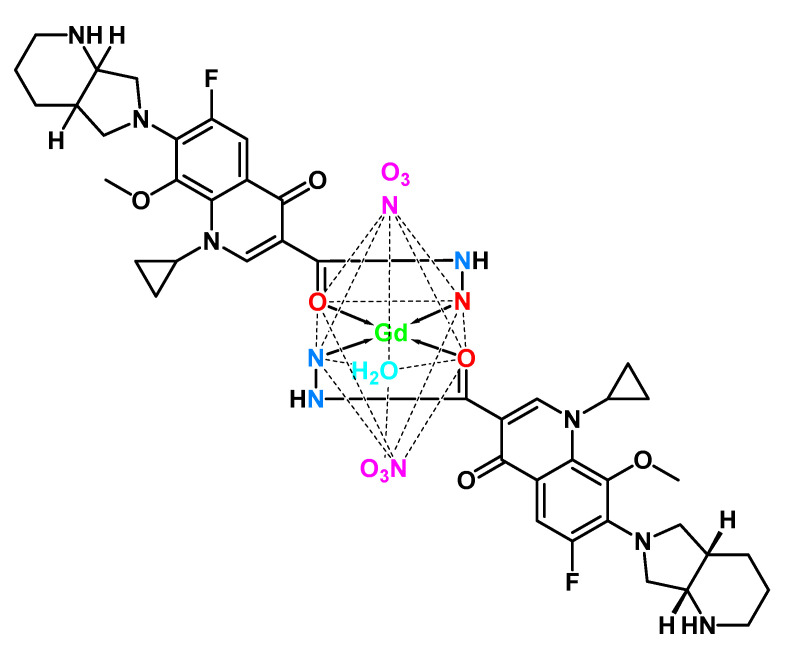
The postulated structures of M(II)MOX-H complexes (M = Co^2+^, Ni^2+^ and Cu^2+^).

Overall, the combined UV–Vis, EPR, magnetic, thermal, conductivity, and elemental analysis data support the proposed molecular formulas and coordination geometries of the MOX-H complexes, as depicted in Figure 3.

### 2.3. Computational Assessment

#### 2.3.1. DFT, Vibrational, Electronic, and Surface Characterization of MOX-H

The structural features of MOX-H were probed theoretically using density functional theory (DFT) in parallel with the experimental FT-IR data. Geometry optimization of the free ligand was carried out at the B3LYP/6-31G(d) level. The calculations indicate that MOX-H is most stable in the amide (keto) tautomer, in which the hydrazide N–H group forms an intramolecular hydrogen bond with the neighboring carbonyl oxygen. This intramolecular H-bonding lowers the energy of the amide form relative to the enol–imine tautomer and is consistent with the broad O–H/N–H stretching features observed experimentally. However, DFT calculations were not performed on the metal complexes. Accordingly, the geometries of the metal complexes discussed herein are based on experimental evidence, supported by the calculated ligand geometry, and should thus be regarded as experimentally inferred rather than quantum-chemically optimized structures.

The simulated vibrational spectrum is in good agreement with the FT-IR data and supports the proposed structure (Figures S32 and S33). The C=O stretching mode appears at a relatively low wavenumber (around 1665 cm^−1^), consistent with hydrogen-bond weakening of the C=O bond. The N–N vibration is calculated in the 1050–1100 cm^−1^ region, while the azomethine C=N stretch is reproduced at 1560–1580 cm^−1^. The close correspondence between calculated and observed bands confirms that MOX-H adopts a hydrogen-bonded amide structure in both the solid state and solution.

**Figure 4:**
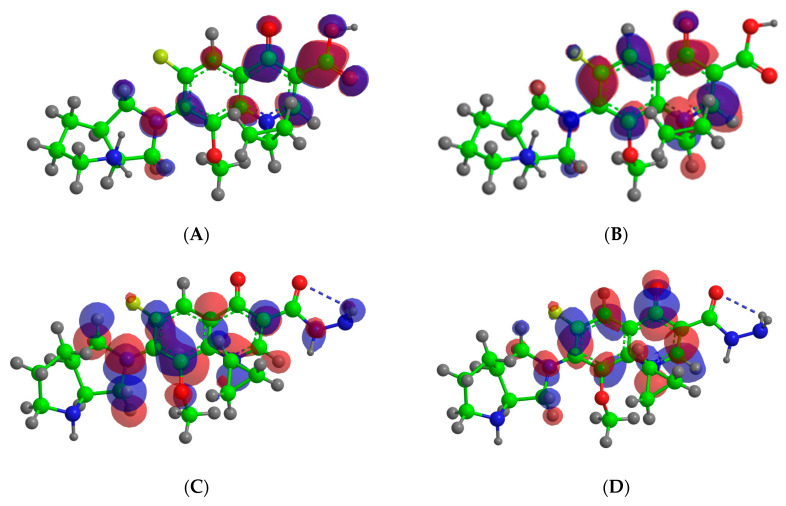
The DFT simulation for the MOX-F (parent drug): (**A**) HOMO and (**B**) LUMO; MOX-H ligand: (**C**) HOMO and (D) LUMO.

To gain further insight into the electronic nature of the ligand, DFT was also used to estimate key electronic parameters such as self-consistent field (SCF) energy, dipole moment, and frontier molecular orbital energies (HOMO and LUMO), as summarized in Figure 4 and Table 3 The optimized MOX-H molecule shows a relatively large dipole moment (11.9 D), reflecting significant polarity that should favor electrostatic and hydrogen-bond interactions with biomolecular targets. The HOMO–LUMO energy separation (Δ*E*) indicates a balance between chemical stability and reactivity, consistent with a molecule capable of efficient intramolecular charge transfer without being overly labile. When compared with the parent drug MOX-HCl (dipole moment 11.83 D), MOX-H displays very similar polarity and a comparable distribution of frontier orbitals, suggesting that the hydrazide modification retains the essential electronic profile of the parent quinolone while adding extra donor/acceptor sites for interaction with biological receptors [32,33].

**Figure 5:**
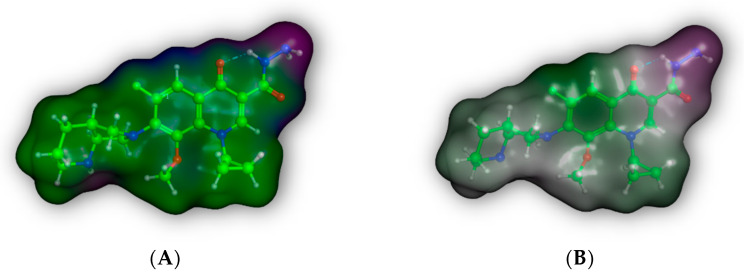
The surface properties of the MOX-H compound, (**A**) = active lone pair map (violet = H-bonding, green = hydrophobic and blue = mild polar), (**B**) = Lipophilicity (violet = hydrophilic, white = neutral and green = lipophilic).

The surface and lipophilic properties of MOX-H were visualized using molecular surface potential and lipophilic maps (Figure 5) to rationalize its behavior in biological media. The lipophilic potential map distinguishes three main surface types: (i) violet regions associated with hydrogen-bond donor/acceptor sites, mainly located on the amide and hydrazide functionalities; (ii) green patches corresponding to hydrophobic areas; and (iii) blue zones representing mildly polar regions. The quinolone scaffold and alkyl substituents are predominantly covered by a green surface, indicating pronounced lipophilicity that is expected to assist in membrane penetration and receptor binding.

The lipophilic contour representation—where violet denotes hydrophilic, white neutral, and green lipophilic fields—shows that MOX-H is characterized by an extended lipophilic envelope interrupted by discrete hydrophilic/H-bonding spots. This pattern points to a molecule capable of interacting with both polar and nonpolar domains: the hydrophilic centers can anchor via hydrogen bonding, while the lipophilic surface promotes partition into lipid environments. Such a combination of high H-bonding capacity and substantial lipophilic character is consistent with the anticipated pharmacological versatility of MOX-H as an active ligand candidate [30,31,32,33].

#### 2.3.2. In Silico Drug Likeness, ADME, and Toxicity

**Figure 6:**
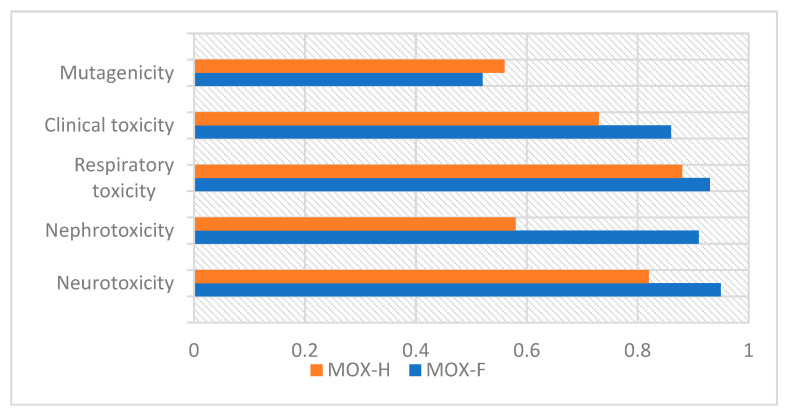
Comparative toxicity probabilities of MOX-F and MOX-H.

Computational properties for the parent MOX-F and its hydrazide analogue MOX-H were obtained using SwissADME, MolSoft, pkCSM, and ProTox 3.0, and they are summarized in Tables S7 and S8; complementary visualizations are provided in Figure S34 (LogP/LogS) and Figure 6 (toxicity probabilities).

Physicochemical profile and oral “rules”: Both analogues satisfy the Lipinski/Veber/Ghose rules. Relative to MOX-F, MOX-H is slightly more lipophilic (LogP 1.69 vs. 1.60) and less soluble (LogS −3.52 vs. −3.43). The hydrazide raises polarity (TPSA 101.62 vs. 83.80 Å^2^) and adds one H-bond donor (HBD 3 vs. 2) while keeping the bioavailability score at 0.55 for both. Importantly, drug-likeness improves from 0.71 (MOX-F) to 0.92 (MOX-H) (Table S8).

Absorption and distribution: Predicted intestinal absorption remains high, albeit lower for MOX-H (84.2%) than MOX-F (93.3%), with similar Caco-2 permeability (1.004 vs. 1.137; tool units). Both are P-gp substrates and not P-gp inhibitors, consistent with controlled efflux. Distribution metrics indicate poor CNS entry in both, with MOX-H showing a more negative CNS permeability (−3.55 vs. −2.65 for MOX-F), matching its higher TPSA. Overall, MOX-H trades a modest decrease in gut uptake for a desirable reduction in CNS exposure.

Safety predictions (quantitative): In Table S7, both compounds are Ames-negative and hERG-inactive; MOX-H shows a higher predicted LD_50_ (3.011 vs. 2.568 mg·kg^−1^) and higher chronic toxicity threshold (1.729 vs. 1.271 mg·kg^−1^·day^−1^)—i.e., lower modelled acute/chronic toxicity than MOX-F. The ProTox probability panel in Table S7 and Figure 2 highlights lower risk probabilities for MOX-H across several endpoints—e.g., neurotoxicity 0.82 vs. 0.95, nephrotoxicity 0.58 vs. 0.91, clinical toxicity 0.73 vs. 0.86, respiratory 0.88 vs. 0.93—with a single immunotoxicity flag for MOX-H (0.95, Active) versus inactive 0.98 for MOX-F. BBB penetration is predicted to be inactive for both.

The physicochemical shifts (slightly higher LogP, higher TPSA) explain the Table S8 ADME outcomes (still-high GI absorption with reduced CNS permeability). The safety panel is satisfactory where it matters the most (mutagenicity and cardiotoxicity), while the improved LD_50_ and lower organ-risk probabilities collectively favor MOX-H. The only cautionary note is the immunotoxicity probability for MOX-H, which prioritizes targeted wet-lab follow-ups (e.g., cytokine-release assays) before in vivo studies. Altogether, the tables and figures converge on the same conclusion: MOX-H retains oral developability (rule-set compliance; bioavailability score 0.55) while improving drug-likeness and lowering predicted CNS exposure and acute toxicity relative to MOX-F, making the hydrazide a more balanced lead for experimental validation.

#### 2.3.3. Molecular Docking Studies

Molecular docking was performed to elucidate the binding behavior of MOX-F, its hydrazide analogue MOX-H, and the Cu–MOX-H complex toward human topoisomerase IIα (Topo IIα). The docking scores demonstrate a clear and progressive improvement in binding affinity along the series: MOX-F < MOX-H < Cu–MOXH, consistent with their structural modifications and experimental biological activities.

Binding Affinity and Energetic Evaluation

MOX-F exhibited the weakest interaction, reflected by its relatively modest docking score (Table 4). Conversion to the hydrazide (MOX-H) significantly improved binding, while complexation with Cu(II) produced the most stable ligand–receptor assembly. The enhanced affinity of MOX-H and its Cu complex is attributed to additional hydrogen bond donors, higher polar surface area, and the presence of a metal center capable of engaging in electrostatic and H–π interactions.

Binding Mode Analysis

The binding poses provide insight into the interaction and visual confirmation of the proposed trends. MOX-F (Figure S36) binds superficially within the DNA-binding cleft of Topo IIα, forming only two weak hydrogen bonds with Glu712 and Lys676. Its orientation lacks significant π-stacking or electrostatic anchoring, explaining the relatively lower docking score and reduced predicted biological impact [11,15,41,42,43,44,45].

**Figure 7:**
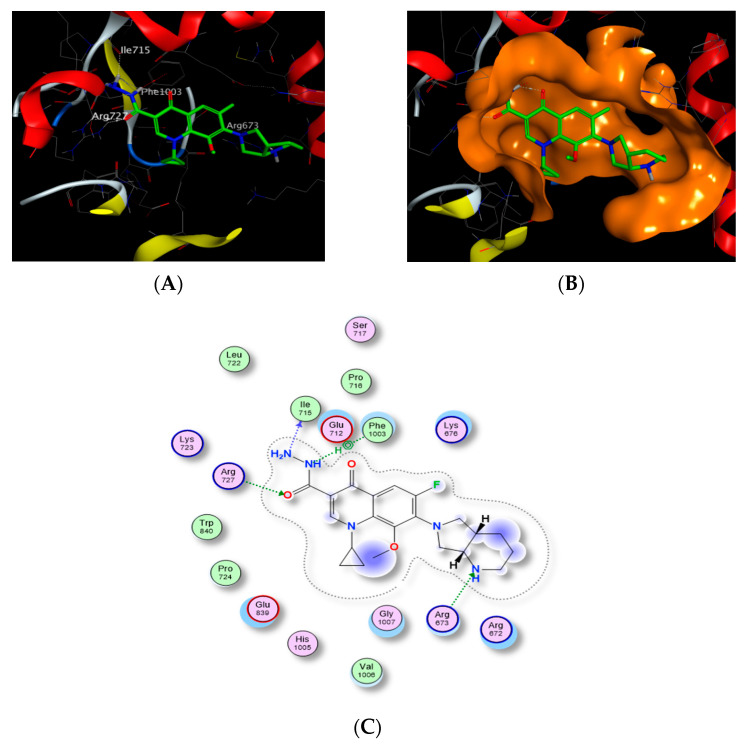
Docking model of the interaction of MOX-H with Topoisomerase II (PDB code: 5GWK): (**A**) 3D interaction diagram, (**B**) Cavity form and (**C**) 2D interaction diagram.

MOX-H (Figure 7) adopts a more embedded and stable pose. The hydrazide moiety promotes a flatter conformation, enabling insertion deeper inside the catalytic pocket. MOX-H forms multiple hydrogen bonds with Ile715, Arg727, and Arg673, along with favorable π–π interactions with DNA bases. This cooperative interaction network strengthens ligand anchoring and better mimics the mechanism by which Topo IIα inhibitors stabilize the DNA–enzyme cleavage complex [46,47,48].

**Figure 8:**
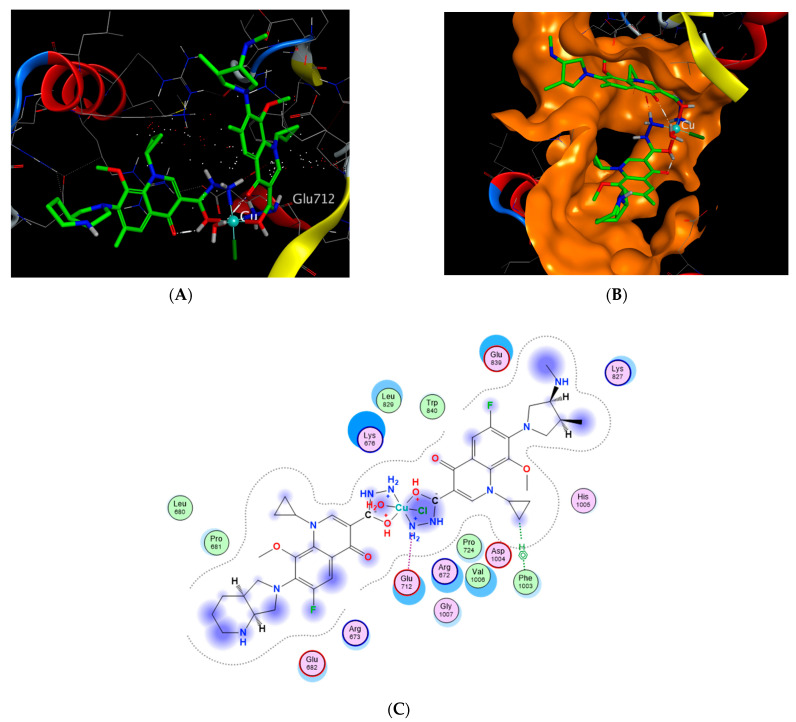
Docking model of the interaction of Cu-MOXH with Topoisomerase II (PDB code: 5GWK): (**A**) 3D interaction diagram, (**B**) Cavity form and (**C**) 2D interaction diagram.

Cu–MOXH (Figure 8) shows the most compact and energetically favorable orientation. The Cu(II) center establishes a strong ionic interaction with Glu712, enhanced by H–π contact with Phe1003. The metal ion drives the entire complex deeper into the active site, creating a dense web of stabilizing interactions. This configuration aligns with the highest docking score and supports the hypothesis that metal coordination enhances enzyme inhibition and may allow for ROS-mediated cytotoxic pathways [47,49,50,51,52,53,54].

Structure–Activity Implications

Across all analyses, docking data consistently favor the hydrazide derivative and its Cu(II) complex: MOX-F is weakly bound, with minimal pocket engagement; MOX-H has improved depth of insertion, richer H-bond network, and DNA-base stacking; and Cu–MOX-H has the strongest binding, metal-assisted interactions, and the most stabilized pose. The docking results also correlate well with physicochemical trends discussed in Tables S7 and S8, where MOX-H exhibits increased polarity and improved drug-likeness, and with experimental cytotoxicity in vitro.

Collectively, the docking scores (Table 4), interaction maps (Figure 7, Figure 8 and Figure S36), and structural analyses agree with the conclusion that hydrazide modification substantially enhances Topo IIα binding, and Cu(II) coordination produces the most potent and structurally coherent inhibitor candidate in the series. These results provide strong mechanistic support for advancing MOX-H, and particularly its Cu(II) complex, into further biological and mechanistic evaluations.

### 2.4. Application of Moxifloxacin Derivatives and Their Complexes

#### 2.4.1. Antimicrobial Activity

**Figure 9:**
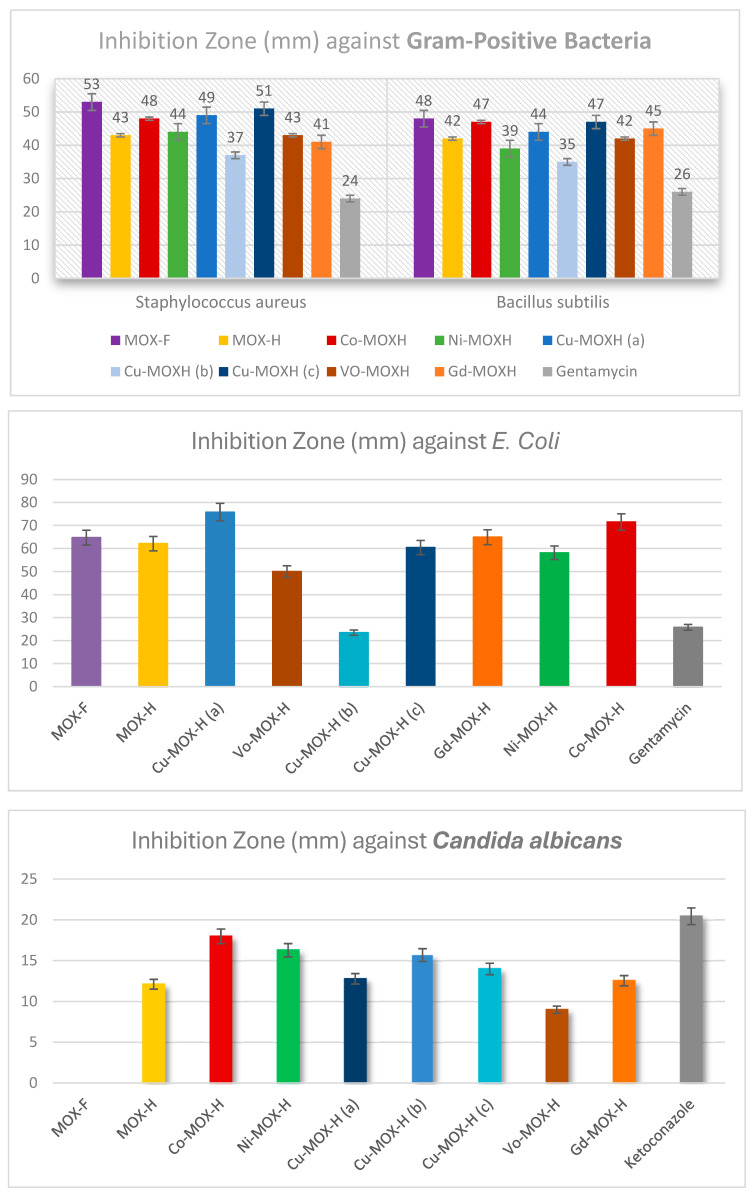
Inhibition zone diameters (mm) of MOX-F, MOX-H, and the synthesized metal complexes against Gram-positive bacteria (*Staphylococcus aureus* and *Bacillus subtilis*), Gram-negative bacteria (*Escherichia coli*), and *Candida albicans*. Results are expressed as mean ± SD $\left(n=3\right)$. Gentamycin and ketoconazole were used as standard antibacterial and antifungal reference drugs, respectively.

The antimicrobial performance of MOX-H and its metal complexes was assessed against representative Gram-positive, Gram-negative, and fungal strains. The full dataset is presented in Tables S9 and S10, with graphic summaries in Figure 9 and Figure S37. The results reveal a consistent and marked enhancement in activity upon metal coordination. Gentamicin and ketoconazole were included as positive controls at their standard as-say concentrations to validate the susceptibility protocol; because these concentrations are not equated on a molar basis to the test complexes, direct equimolar ranking versus the controls is not claimed.

The free hydrazide MOX-H exhibits moderate antimicrobial potency; however, coordination to Cu(II), Co(II), and Ni(II) dramatically strengthens activity across all organisms. Against *S. aureus* and *B. subtilis*, these complexes produced inhibition zones of 48–51 mm, substantially exceeding both MOX-H and the reference drug gentamycin. Even more striking was the fact that inhibition of *E. coli* reached 70–75 mm, which is among the largest zones reported for moxifloxacin-derived metal complexes. Correspondingly, MIC values dropped from 3008 μM for MOX-H to 76 μM for the best-performing complexes, indicating a greater than tenfold increase in potency. Antifungal activity shows a similar trend. MOX-H exhibits modest inhibition of *C. albicans* (12 mm), whereas coordination to Cu(II) or Co(II) increases antifungal activity to 16–18 mm, approaching ketoconazole (20 mm). Inhibition-zone diameters are used here as qualitative screening indicators under fixed mass loading and are influenced by diffusion/solubility; therefore, they are not treated as equimolar potency comparisons to the reference drugs. Quantitative comparisons are based primarily on MIC values expressed in µM.

Fluoroquinolones, including moxifloxacin, are well documented to chelate metal ions in aqueous environments; potentiometric studies report formation of moxifloxacin complexes with Co(II), Ni(II), and Cu(II) in aqueous electrolyte mixtures, confirming that coordination is chemically viable in water [55]. However, the extent to which a given solid-state formulation remains intact upon dilution depends on pH, ionic strength, concentration, and competition from other ligands/ions. Reviews of fluoroquinolone–metal chemistry emphasize that speciation can shift under biologically relevant conditions and therefore bioactivity should be interpreted cautiously when direct solution-stability measurements are not available [56]. Accordingly, the antimicrobial results reported here are discussed as complex-derived activity under the assay conditions, consistent with an equilibrating metal–ligand system rather than attributed exclusively to a single intact complex species.

The consistent superiority of the complexes can be rationalized by standard properties of metal complexes: metal coordination increases lipophilicity, facilitates membrane permeation, and polarizes the ligand framework, enabling stronger interactions with microbial proteins and DNA. Taken together, the antimicrobial dataset demonstrates that metal coordination transforms MOX-H from a moderately active agent into a broad-spectrum, highly potent candidate [57,58,59].

#### 2.4.2. Cytotoxicity and Selectivity

The anticancer activity of MOX-H and the Cu–MOX-H complex was evaluated against MCF-7 and HepG2 cancer cells, with Vero cells used to assess selectivity. The results are compiled in Table S11 and visualized in Figure 10 and Figures S38–S47.

MOX-H significantly improves upon the parent drug (MOX-F), reducing the IC_50_ value in MCF-7 cells from 69.5 μM to 8.61 μM, and in HepG2 from 45.3 μM to 30.4 μM, while maintaining lower toxicity toward normal Vero cells. These trends are reflected in improved selectivity indices (up to 4.96), suggesting that hydrazide conversion enhances targeting of malignant cells.

Coordination with Cu(II) further amplifies these effects. The Cu–MOX-H complex displays the highest potency (MCF-7 IC_50_ = 7.50 μM; HepG2 IC_50_ = 16.96 μM) while retaining acceptable selectivity toward normal cells. The improved activity is consistent with literature reports indicating that Cu(II) complexes can combine DNA interaction with redox activity; however, no direct ROS assay was performed in the present study, and any ROS-mediated contribution should therefore be regarded as a plausible mechanistic hypothesis rather than an experimentally established pathway of action [60,61,62].

These findings highlight a synergistic enhancement: hydrazide derivatization improves molecular recognition, while Cu(II) coordination boosts redox-driven cytotoxic pathways. The combined effect places Cu–MOX-H among the most biologically active and selective moxifloxacin-derived metal complexes reported to date.

Across all antimicrobial and cytotoxic assays, the data converge on a clear structure–activity pattern: MOX-H improves potency relative to MOX-F due to increased hydrogen-bonding capacity and altered polarity. Metal coordination—especially with Cu(II), Co(II), and Ni(II)—further enhances activity, reducing MIC values and lowering IC_50_ values. Cu–MOX-H emerges as the lead candidate, combining potent antimicrobial effects, nanomolar–micromolar anticancer activity, and acceptable selectivity. The Co(II), Ni(II), and Cu(II) series provide a useful horizontal comparison across the late first-series transition metals, with a smooth variation in metal *d* electron count: *d*^7^, *d*^8^, and *d*^9^, respectively. The VO(IV) and Gd(III) complexes provide useful comparisons outside of this series, corresponding to a more highly oxidized, early transition metal and a lanthanide, respectively.

Within the limited metal series examined here, the Co(II), Ni(II), and Cu(II) complexes provide a useful comparative trend in biological activity; however, this observation should be regarded as system-specific and preliminary rather than as a general correlation with d-electron count. Broader confirmation would require a more extensive series, including complexes of additional first-row transition metals such as Mn and Fe, under comparable conditions. Future work may therefore extend this ligand platform to additional metal ions, including Zn(II), Mn(II), and Fe(III/II), in order to test whether the activity trends observed here persist across a broader and more systematic series.

The results underscore the strong potential of hydrazide–metal frameworks as multifunctional antibacterial and anticancer scaffolds, justifying their advancement to mechanistic and in vivo studies.

### 2.5. Integrated Computational–Biological Insight

Our computational work helps make sense of why MOX-H and its metal complexes behave so well in the biological assays. The ADMET predictions showed that converting MOX-F into the hydrazide form gives the molecule a better balance of polarity and lipophilicity, improves drug-likeness, and lowers predicted toxicity. These shifts match perfectly with the stronger antibacterial and anticancer activities we observed in vitro. Docking studies reinforced this trend, revealing that MOX-H—and especially the Cu–MOX-H complex—binds more tightly and more favorably to Topoisomerase IIα than the parent drug. In short, the computational models anticipated the biological improvements we later confirmed experimentally.

The electronic structure data and spectroscopic results add another layer of understanding. They show that coordination with metals increases charge polarization and strengthens interactions within the ligand framework, which helps explain why the Cu(II), Co(II), and Ni(II) complexes consistently outperformed the free hydrazide. The Cu complex, in particular, benefits from both strong binding interactions and useful redox activity, giving it an advantage in penetrating cells and damaging microbial and cancer targets. These combined features align with its superior MIC and IC_50_ values across the entire study. Altogether, the computational and experimental findings point to Cu–MOX-H as a thoughtfully designed, biologically validated lead worth advancing to the next stages of development.

The precise solution stability and speciation of the complexes under physiological conditions were not directly quantified in the present study; therefore, partial dissociation into ligand- and metal-containing species in biological media cannot be excluded. Accordingly, the observed biological effects are interpreted as arising from the metal–ligand system present under the assay conditions rather than from a single confirmed molecular species, and bioactive complexes here described may be functioning as prodrugs.

## 3. Materials and Methods

### 3.1. Chemicals, Instrumentation, Physical Measurement, and Calculations

Sigma-Aldrich supplied all analytical-grade chemical reagents, which were used without additional purification. A licensed pharmaceutical manufacturer provided the pure form of moxifloxacin.

The instrumentation and physical measurements used for compound characterization, thermal analysis, magnetic susceptibility, mass spectrometry, X-ray diffraction, and spectroscopic evaluations (FT-IR, NMR, UV-visible, EPR) are detailed in the Supplementary Information.

Prior to analysis, the isolated ligand and complexes were carefully dried and stored under closed conditions; minor deviations between found and calculated elemental values are therefore attributed to trace retained moisture/solvent and the hydrated nature of the isolated solids.

### 3.2. Synthesis

#### 3.2.1. Moxifloxacin Hydrazide (MOX-H)

**Scheme 1:**
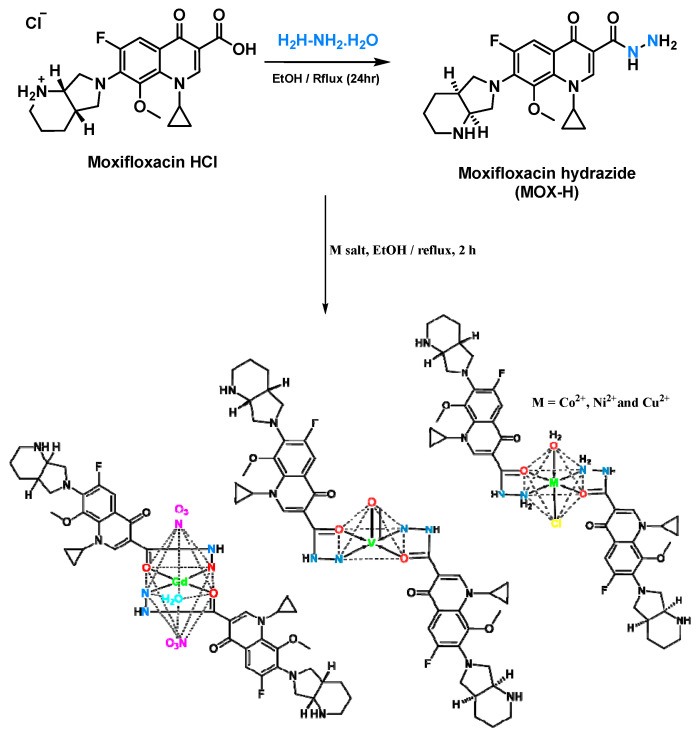
Synthetic route for the preparation of moxifloxacin hydrazide (MOX-H) from moxifloxacin (MOX-F) and the subsequent synthesis of the corresponding Co, Ni, Cu, VO(IV), and Gd complexes in ethanolic medium under reflux.

After dissolving 4.37 g (10 mmol) of moxifloxacin hydrochloride in 25 mL of 80% hydrazine hydrate, the mixture was heated at reflux under atmospheric pressure for 24 h. The mixture was left to cool to room temperature once the reaction was finished. To obtain a brownish-buff crystalline powder, the resultant solid was filtered, washed, recrystallized from ethanol, and vacuum-dried. The product was stored in a desiccator until further use. The yield was approximately 76%, and the product had a melting point of 222 °C. The structure was confirmed by spectroscopic and other analytical techniques (Scheme 1).

#### 3.2.2. Synthesis of Metal Complexes

Metal complexes of MOX-H were synthesized by mixing hot ethanolic solutions of metal salts (0.1 mmol, 20mL) with the ligand (MOX-H, 0.2 mmol, 0.083 g) dissolved in 20 mL of ethanol. The metal salts used included CoCl_2_·6H_2_O (0.0238 g), NiCl_2_·6H_2_O (0.0237 g), CuCl_2_·2H_2_O (0.017 g), VOSO_4_·5H_2_O (0.024 g), and Gd(NO_3_)_3_·6H_2_O (0.0541 g).

The metal solution was added dropwise to the ligand solution under reflux with continuous stirring for 3 h. Upon completion of the reaction, the mixtures were cooled and the resulting solid precipitates were filtered, washed with cold ethanol, and air-dried. The products were obtained as colored solid complexes characteristic of each metal ion, with isolated yields ranging from 65–85% and were stored in airtight containers prior to characterization. Repeated attempts to obtain single crystals suitable for X-ray diffraction under different crystallization conditions were unsuccessful.

The Cu complex was synthesized using two alternative methods: microwave-assisted synthesis and hydrothermal synthesis. For the microwave-assisted route, MOX-H (0.2 mmol) and copper(II) chloride (0.1 mmol) were mixed in ethanol (20 mL) in a beaker, then irradiated in a microwave reactor at 600 W for 2 min. After completion, the resulting solid was isolated by filtration, washed, and dried under vacuum. For the hydrothermal route, MOX-H (0.2 mmol) and copper(II) chloride (0.1 mmol) were dissolved in ethanol (20 mL), and the solution was transferred to a Teflon-lined stainless-steel autoclave. The sealed autoclave was heated at 120 °C for 6 h and then allowed to cool to room temperature. The product was collected by filtration and dried in a desiccator.

### 3.3. In Silico Predictions of Physicochemical Properties, Pharmacokinetics, and Bioactivity

Geometry optimization of the free ligand MOX-H was carried out at the B3LYP/6-31G(d) level using Maestro 14.3. Vibrational-frequency calculations were used to confirm that the optimized structure corresponds to a true minimum. DFT calculations in the present study were restricted to the free ligand MOX-H and were used to support its geometry, tautomeric preference, vibrational interpretation, and frontier-orbital properties. No DFT geometry optimization was performed for the metal complexes, because these open-shell transition-metal and lanthanide systems require a more specialized computational treatment beyond the scope of the present work. Their coordination geometries are therefore proposed from the combined experimental evidence.

Molecular surface analysis and MM2 molecular mechanics calculations were conducted using Avogadro and Chem3D Ultra, respectively. The total steric energy, torsional strain, and van der Waals interactions were computed to identify the most stable conformers of MOX-H. In addition to DFT optimization, conformational analysis of MOX-H was carried out using MM2 molecular mechanics in order to identify the lowest-energy conformer prior to discussion of the optimized ligand structure.

AutoDock Vina 1.2.3, an open-source platform, was used for molecular docking, targeting human DNA topoisomerase IIα (PDB ID: 5GWK). Ligand structures were prepared using Open Babel, and receptor preparation (adding hydrogens, removing water, assigning charges) was done via AutoDock Tools 1.5.7. PyMOL and Discovery Studio Visualizer were used to assess the docking results after a grid box was constructed to include the complete active site of the enzyme.

ADMET properties and drug-likeness predictions were assessed using freely accessible web tools: SwissADME (http://www.swissadme.ch/, accessed on 1 April 2024) for lipophilicity, TPSA, and oral bioavailability; MolSoft for drug-likeness scoring; and pkCSM and ProTox-II for pharmacokinetics and toxicity predictions [63,64,65].

### 3.4. Pharmacology and Biology

#### 3.4.1. In Vitro Antibacterial/Antifungal Evaluation

(MOX-H) derivatives, along with their related metal complexes, were investigated in vitro against a panel of representative microbial strains. The assessment was carried out employing the agar well diffusion technique in parallel with the determination of minimum inhibitory concentration (MIC) values. The examined microorganisms comprised *Staphylococcus aureus* (ATCC 25923), *Bacillus subtilis* (RCMB 015 (1) NRRL B-543), *Escherichia coli* (ATCC 25922), and *Candida albicans* (RCMB 005,003 (1) ATCC 10231).

The antimicrobial assays were performed using freshly prepared solutions of the complexes under the stated assay conditions. Because fluoroquinolone-type ligands can undergo condition-dependent coordination equilibria with metal ions in aqueous media and because biological media contain competing ligands/ions—the precise solution speciation (intact complex vs. partially dissociated forms) was not independently quantified in this work. Therefore, biological outcomes are interpreted as complex-derived activity under assay conditions, reflecting the metal–ligand system present in solution rather than a single confirmed molecular species [55].

Stock solutions of the tested compounds were freshly prepared in distilled water to obtain a final concentration of 20 mg/mL. Nutrient agar plates were aseptically inoculated with the corresponding microbial suspensions, after which circular wells of 6 mm diameter were carefully made in the solidified agar. Subsequently, 100 μL of each test solution was introduced into the designated wells. Gentamicin (4 mg/mL) and ketoconazole (100 μg/mL) were employed as reference drugs for antibacterial and antifungal activity, respectively. The inoculated plates were incubated at 37 °C for 24 h in the case of bacterial strains, whereas fungal cultures were maintained at 28–30 °C for 48 h. Antimicrobial effectiveness was quantitatively estimated by measuring the diameters of the inhibition zones, expressed in millimeters, including the well diameter. For MIC determination, a series of successive dilutions of each compound was prepared in distilled water and tested under identical incubation conditions.

The MIC value was recorded as the minimum concentration at which no visible microbial growth could be observed. All experiments were performed in triplicate to ensure reproducibility and reliability of the obtained results [14,66,67].

#### 3.4.2. In Vitro Cytotoxicity Assay (MTT)

The cytotoxic activity of the tested samples was evaluated using mammalian cell lines: Vero (normal), MCF-7 (human breast adenocarcinoma), and HepG2 (human hepatocellular carcinoma). Cells were obtained from a certified cell bank and routinely cultured in RPMI-1640 medium supplemented with 10% fetal bovine serum (FBS), 100 U/mL penicillin, and 100 μg/mL streptomycin. Cells were maintained at 37 °C in a humidified atmosphere containing 5% CO_2_, subcultured upon reaching approximately 80–90% confluence, and only cells in the exponential growth phase were used for viability and morphological assessment.

For the MTT assay, cells were seeded in 96-well plates at a density of 1 × 10^5^ cells/mL (100 μL per well) and incubated at 37 °C and 5% CO_2_ for 24 h to allow monolayer formation. The growth medium was then aspirated, and the cell monolayer was washed twice with sterile phosphate-buffered saline (PBS). Tested samples were prepared as two-fold serial dilutions in RPMI-1640 supplemented with 2% FBS (maintenance medium), and 100 μL of each dilution was added to the corresponding wells. Control wells received maintenance medium only (three wells per plate). Cells were incubated with the tested compounds for 24 h prior to MTT addition, and this exposure time was identical for all tested cell lines. During incubation, cells were examined under an inverted microscope for morphological indicators of cytotoxicity (e.g., rounding, shrinkage, granulation, or partial/complete loss of the monolayer).

After the 24 h exposure period, 20 μL of MTT reagent (5 mg/mL in PBS) was added to each well, plates were shaken at 150 rpm for 5 min to ensure uniform distribution and then incubated at 37 °C and 5% CO_2_ for an additional 4 h to allow formazan formation. The supernatant was removed, and the resulting formazan crystals were dissolved in 200 μL dimethyl sulfoxide (DMSO) with shaking (150 rpm, 5 min). Absorbance was measured at 560 nm with background subtraction at 620 nm. Cell viability (%) was calculated relative to untreated controls using background-corrected absorbance values [68,69,70].

IC_50_ values were determined from the concentration–viability relationship by identifying the concentration that reduced viability to 50% of the untreated control using standard dose–response analysis

## 4. Conclusions

In this work, we synthesized and characterized moxifloxacin hydrazide (MOX-H) and a representative set of its metal complexes spanning first-row divalent ions (Co(II), Ni(II), Cu(II)), oxovanadium(IV) (vanadyl), and Gd(III), integrating spectroscopic, thermal, magnetic, and computational analyses to rationalize their coordination modes and physicochemical behavior. The combined DFT, ADMET, and molecular docking studies provided a coherent mechanistic picture, showing that hydrazide formation enhances the molecular properties of moxifloxacin. At the same time, metal coordination, particularly with Cu(II), further refines electronic structure, increases binding affinity toward Topoisomerase IIα, and improves predicted drug-likeness and safety. These computational insights were strongly supported by the biological findings: MOX-H and its metal complexes exhibited markedly enhanced antimicrobial potencies, with significantly reduced MIC values, and demonstrated promising anticancer activity with favorable selectivity profiles. Among the tested complexes, Cu–MOX-H consistently emerged as the most active agent, reflecting a synergistic interplay between hydrazide derivatization and copper-mediated redox and binding mechanisms. Together, these results highlight the value of combining ligand modification with metal coordination to achieve multifunctional therapeutics and position Cu–MOX-H as a compelling candidate for further mechanistic exploration and preclinical evaluation.

## Figures and Tables

**Figure 10:**
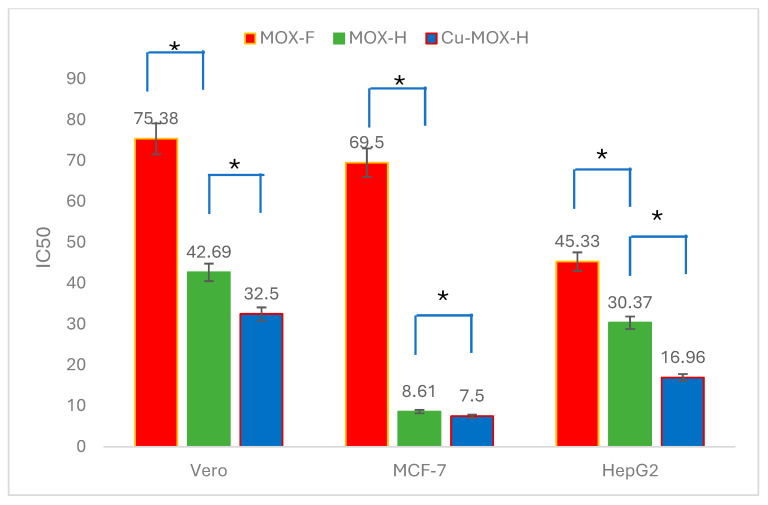
${IC}_{50}$ values of MOX-F, MOX-H, and Cu-MOXH against Vero, MCF-7, and HepG2 cell lines. Data are presented as mean ± SD $\left(n=3\right)$. Statistical analysis was performed using one-way ANOVA. * Statistically significant difference at p<0.05.

**Table 1:** Analytical data and conductivity measurements of MOX-H and its complexes.

Compound	Molecular Weight	Color	Melting Point (°C)	Ω ^‡^	M%		
					Found		Calc.
					A	B	
MOX-H	415.47	Brownish buff	220	-	-	-	-
[Co(H_2_O)Cl(MOX-H)_2_]Cl·2.5H_2_O	1023.84	Dark brown	280	53	5.45	5.23	5.76
[Ni(H_2_O)Cl(MOX-H)_2_]Cl·4.5H_2_O	1059.64	Dark green	>300	50	5.80	6.01	5.53
[Cu(H_2_O)Cl(MOX-H)_2_]Cl·2H_2_O (a)	1019.44	Dirty green	208	40	6.42	5.85	6.23
[Cu(H_2_O)Cl(MOX-H)_2_]Cl·2.5H_2_O (b)	1028.46	Dirty green	180	42	4.31	3.92	4.20
[Cu(H_2_O)Cl(MOX-H)_2_]Cl·0.5H_2_O (c)	992.42	Dirty green	130	45	8.78	8.32	8.87
[VO(MOX-H)_2_]SO_4_·3.5H_2_O	1056.99	Brick brown	>300	40	-	5.10	4.81
[Gd(H_2_O)(NO_3_)_2_(MOX-H)_2_]NO_3_·2H_2_O	1228.27	Greenish yellow	218	97	-	12.27	12.80

^‡^ 10^−3^ M in DMSO, ohm^−1^ cm^2^ mol^−1^, A = Complexometric titration, B = Thermo-gravimetric analysis; (a) Template method. (b) Microwave method. (c) Hydrothermal method.

**Table 2:** Significant IR frequencies (cm^−1^) for moxifloxacin hydrazide and its complexes.

Compound	υ (OH, H_2_O, NH, NH_2_)	υ (C=O)	υ (C=O) Quinolone	υ (NH) Bending	υM-O	υM-N
MOX-F·HCl	3530–3471 (br,m)	1708 (s)	1623 (s)	-	-	-
MOX-H	3430–3238 (br,m)	1665 (s)	1621 (m)	1568 (m)	-	-
[Co(MOX-H)_2_(H_2_O)Cl]Cl·2.5H_2_O	3422 (br,s)	-	1621 (m)	1564 (w)	712	619
[Ni(MOX-H)_2_(H_2_O)Cl]Cl·4.5H_2_O	3418 (br,s)	-	1621 (m)	1559 (w)	720	617
[Cu(MOX-H)_2_(H_2_O)Cl]Cl·2H_2_O (a)	3425 (br,s)	-	1621 (m)	1574 (w)	720	619
[Cu(MOX-H)_2_(H_2_O)Cl]Cl·2.5H_2_O (b)	3433 (br,s)	-	1619 (m)	1563 (w)	720	619
[Cu(MOX-H)_2_(H_2_O)Cl]Cl·0.5H_2_O (c)	3423 (br,s)	-	1621 (m)	1573 (w)	720	617
[VO(MOX-H)_2_]SO_4_·3.5H_2_O	3422(br,s)	1655 (sh)	1621 (m)	1565 (w)	714	619
[Gd(MOX-H)_2_(H_2_O)(NO_3_)_2_]NO_3_·2H_2_O	3422 (br,s)	1649 (w)	1620 (m)	1574 (w)	714	617

s: strong, m: medium, w: weak, sh: shoulder, br: broad. (a) Template synthesis (b) microwave-assisted synthesis (c) hydrothermal synthesis.

**Table 3:** The DFT simulation data and ligand properties of MOX-F and MOX-H.

Compound		MOX-F	MOX-H
Formula		C_21_H_24_FN_3_O_4_	C_21_H_26_FN_5_O_3_
Atoms		54	56
Electrons		213	220
Self-consistent field energy (SCF)	(au)	−1372.29	−1407.02 au
	(kJ/mol)	3.60 × 10^6^	3.69 × 10^6^
Dipole		11.83 D	11.9 D
E_LUMO_		−0.068 au	−0.065 au
E_HOMO_		−0.083 au	−0.179 au
ΔE_HOMO-LUMO_		139.17 nm	137.87 nm
Ionization potential (I)		0.083 au	0.179 au
electron affinity (A)		0.068 au	0.06 5au
Absolute electronegativity (χ)		0.075 au	0.122
Absolute hardness (η)		0.0075 au	0.057
Absolute softness (σ)		133.33 au^−1^	17.54
Global softness (S)		66.67 au^−1^	8.77
Global electrophilicity (ω)		0.38 au	0.130
Chemical potential (Pi)		−0.075 au	−0.122
Additional electronegativity (ΔN_max_)		10.06	2.14

**Table 4:** Molecular docking scores, RMSD values, binding free energies, and key ligand–residue interactions of MOX-F, MOX-H, and Cu-MOXH in the active site of human topoisomerase IIα.

Compounds	Score (kcal/mol)	RMSD (A°)	Bonds Between Atoms of Compounds and Residues of Active Site					Total Free Binding Energy (kcal/mol)
			Compound Atom	Receptor Atom	Type of the Interaction	Distance (A°)	Binding Energy (kcal/mol)	
MOX-F	−6.8	1.36	C 13	O (Gly 1007)	Backbone donor (H-donor)	3.29	−0.5	−12.2
			N 36	O (Glu 712)	Backbone donor (H-donor)	3.12	−1.2	
			O 25	N (Lys 676)	Sidechain acceptor (H-acceptor)	3.34	−2.3	
MOX-H	−7.83	0.96	N 54	O (Ile 715)	Backbone donor (H-donor)	2.98	−0.5	−12.19
			O 24	NH (Arg 727)	Sidechain acceptor (H-acceptor)	3.14	−0.8	
			O 24	NH_2_ (Arg 727)	Sidechain acceptor (H-acceptor)	3.09	−1.3	
			N 36	N (Arg 673)	Sidechain acceptor (H-acceptor)	3.21	−2.9	
			N 22	(Phe 1003)	H-Arene (H-pi)	4.28	−0.6	
Cu-MOXH	−9.07	1.51	N 65	O (Glu 712)	Ionic	3.22	−3.2	−10.19
			C 46	6-Ring (Phe 1003)	H-pi	4.65	−0.5	

## Data Availability

The original contributions presented in this study are included in the article and Supplementary Material. Further inquiries can be directed to the corresponding author.

## Supplementary Materials

The following supporting information can be downloaded at: https://www.mdpi.com/article/10.3390/ijms27073057/s1.
