# Supplementary material for: Design, Synthesis, and Bioevaluation of Moxifloxacin Hydrazide Metal Complexes: Integrated Spectroscopic, Computational, Antimicrobial, and Anticancer Investigations
Source: Int J Mol Sci. 2026 Mar 27;27(7):3057. doi: 10.3390/ijms27073057 (PMC13073143; doi:10.3390/ijms27073057)
Supplement: Supplementary file 1 [file ijms-27-03057-s001.zip › ijms-4205226-supplementary.pdf]

International Journal of *Molecular Sciences*

MDPI

Supplementary

Design, Synthesis, and Bioevaluation of Moxifloxacin Hydrazide Metal Complexes: Integrated Spectroscopic, Computational, Antimicrobial, and Anticancer Investigations

Abbas Mamdoh Abbas [1,*], Sara Reda Fisal [1], Ibrahim A. I. Ali [1], Warren Christopher Boyd [2], Haitham Kalil [3,*] and Adel Sayed Orabi [1]

[1] Department of Chemistry, Faculty of Science, Suez Canal University, Ismailia 41522, Egypt; sarareda@science.suez.edu.eg (S.R.F.); ibrahim3369@yahoo.com (I.A.I.A.); orabiadel@hotmail.com (A.S.O.)

[2] Department of Chemistry, College of Arts and Sciences, Cleveland State University, Cleveland, OH 44115, USA; w.c.boyd59@csuohio.edu

[3] Department of Chemistry & Biochemistry, California Polytechnic State University, San Luis Obispo, CA 93407, USA

* Correspondence: abbasmamdoh@science.suez.edu.eg (A.M.A.); hkalil@calpoly.edu (H.K.)

Chemicals, Instrumentation, Physical Measurement, and Calculations

All chemical reagents were of analytical grade and obtained from Aldrich. Moxifloxacin, in its pure form, was sourced from a Pharmaceutical Corporation and used without additional purification. Cupric chloride dihydrate ($CuCl_2{\cdot}2H_2O$), Nickel chloride hexahydrate ($NiCl_2{\cdot}6H_2O$), Cobalt chloride hexahydrate ($CoCl_2{\cdot}6H_2O$), Vanadyl sulfate pentahydrate ($VOSO_4{\cdot}5H_2O$), and Gadolinium nitrate hexahydrate ($Gd(NO_3)_3{\cdot}6H2O$) were acquired from Aldrich. Absolute ethanol was used as a solvent for the synthesis. Double-distilled water was used to prepare the solutions, and distilled water was employed to rinse the required apparatus, which were dried in the oven before use.

Instrumentation and Physical Measurements

Melting points of the synthesized compounds were measured in open capillaries using an electrical melting point apparatus. The molar conductance of freshly prepared $1{\times}10^{-3}$ M DMSO solutions of the solid complexes was recorded at room temperature with a WTW digital conductivity meter. Metal contents were analyzed by complexometric titration with EDTA using murexide indicator at pH 10 (ammonia buffer). The ^{1}H NMR spectra of the Schiff base ligand were recorded on a Bruker Avance III 400 MHz spectrometer at room temperature in DMSO-d_6, using tetramethylsilane (TMS) as the internal standard; chemical shifts are reported in δ ppm. The electronic absorption spectra of the complexes were recorded on a Shimadzu UV-1800 double-beam spectrophotometer over 200–800 nm, using analytical-grade methanol as the solvent and a 1 cm path-length quartz cell. FT-IR spectra were recorded on a Bruker Tensor 27 spectrophotometer using KBr discs in the 4000–400 cm^{-1} range. Mass spectra were obtained on a Thermo Scientific GC-MS model ISQ instrument. Magnetic susceptibility measurements were performed at room temperature using the modified Gouy method with mercury(II) tetrathiocyanatocobaltate(II) as a calibrant. Elemental analyses (C, H, N) were conducted on a Heraeus CHN-rapid analyzer. Thermal analyses (TGA, DTG, and DTA) were performed using a Shimadzu 60 thermal analyzer under a continuous nitrogen flow (40 mL min^{-1}) at a heating rate of 10 °C min^{-1} across the temperature range of 40–800 °C.

Molecular modeling and energy optimization of the ligand and its complexes were performed using the MM2 force field in ChemOffice.

 https://doi.org/10.3390/ijms27073057

Calculations

Kinetic and thermodynamic parameters

In order to assess the influences of the structural properties of the ligand on thermal behavior of the complexes, the order n, the pre-exponential factor Z and the heat of activation ΔE_a of the various decomposition stages were determined from the TG and DTG thermograms using the Coats-Redfern equation . The approach of computation centred on the following two equations:

$$\ln\left[\frac{1-(1-\alpha)^{1-n}}{(1-n)T^2}\right]=\frac{M}{T+B} \quad \text{for } n\pm1 \qquad \textbf{(S1)}$$

$$\ln\left[\frac{-\ln(1-\alpha)}{T^2}\right]=\frac{M}{T+B} \quad \text{for } n=1 \qquad \textbf{(S2)}$$

Where M = - E/R and B = ln ZR/ØE; **Z** = the pre-exponential factor, **R** = the gas constant, **Ø** = the heating rate, **E** = activation energy and **α** = fraction of the reactant decomposed at time t

The activation entropy **ΔS***, the activation enthalpy **ΔH*** and the free energy of activation **ΔG***were calculated using the following equations:

$$\Delta \mathbf{S}^*= 2.303\left[\frac{\log Zh}{k\,T}\right]R \qquad (S3)$$

$$\Delta \mathbf{H}^*= \Delta E_a - RT \qquad (S4)$$

$$\Delta \mathbf{G}^*= \Delta H^* - T\Delta S^* \quad (S5)$$

Where **k** and **h** are the Boltzmann's and Plank's constants.

Magnetic Moment and UV-Vis Spectra

The measurements were carried out using Sherwood Scientific's Magnetic Susceptibility Balance.

The mass magnetic susceptibility (χ_g) was calculated by:

$$\chi_g = \frac{[\mathbf{LC(R-Ro)}]}{[\mathbf{M\,x10^9}]}$$

Where:

L = sample length in centimeters

M = sample mass in grams

C = balance calibration constant (printed on the back of the instrument)

R = reading from the digital display when the sample (in the sample tube) is in the balance.

$\mathbf{R_o}$ = reading from the display when the empty sample tube is in the balance.

The magnetic properties are due to the presence of unpaired electrons in the partially filled d-orbital in the outer shell of these elements. The number of unpaired electrons were evaluated by magnetic susceptibility, where paramagnetic metal complexes gave magnetic moment from the spins of unpaired electrons.

The effective magnetic moment value of metal complex, μ_{eff}, was given by the sum of spin moments:

$$\mu_{eff} = \mu_{spin} = \sqrt{4s(s+1)} = \sqrt{n(n+2)}$$

Where spin moments **S = 1/2n** and **n** is the number of unpaired electrons .

https://doi.org/10.3390/ijms27073057

Also, the effective magnetic moment was calculated from the molar susceptibility by the following equation:

$$\mu eff = 2.83\sqrt{X_M . T}$$

Where, χ_M is the molar susceptibility which equal mass susceptibility multiplied by mole weight and **T** is the absolute temperature.

The UV- vis absorption spectra which produced due to the electronic transitions from the highest occupied molecular orbital (HOMO) to the lowest unoccupied molecular orbital (LUMO) ,were carried out in water.

X-Ray Diffraction Analysis

The average particle size (ζ) can be calculated from the XRD pattern according to Debye-Scherrer equation:

$$\zeta = \frac{K\lambda}{\beta_h cos\theta}$$

Where:

λ= the wavelength of X-ray radiation (1.541874Å)

K= constant taken as 0.95 for organic compounds

β_h= width at half maximum of the peak measured in radians

θ =position of the working peak which has β_h

From the obtained **λ**, the dislocation density (**δ**) obtained which identify the number of dislocation lines per unit area of the crystal, which can be calculated using the following equation:

$$\delta = \frac{1}{\zeta^2}$$

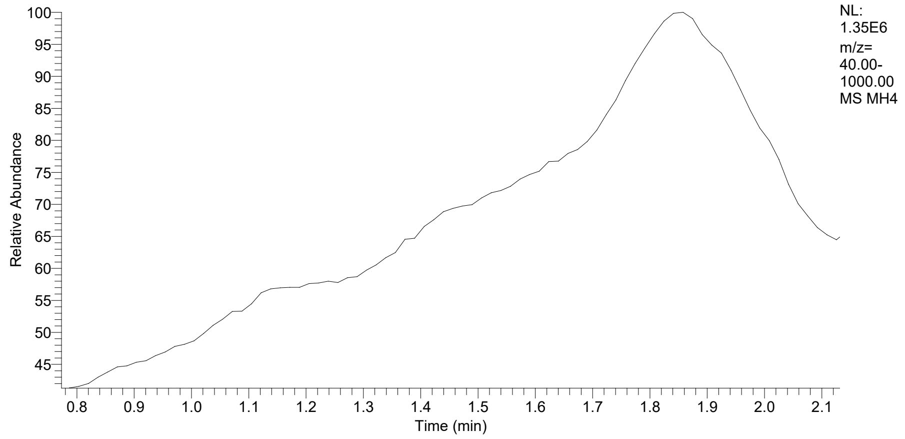

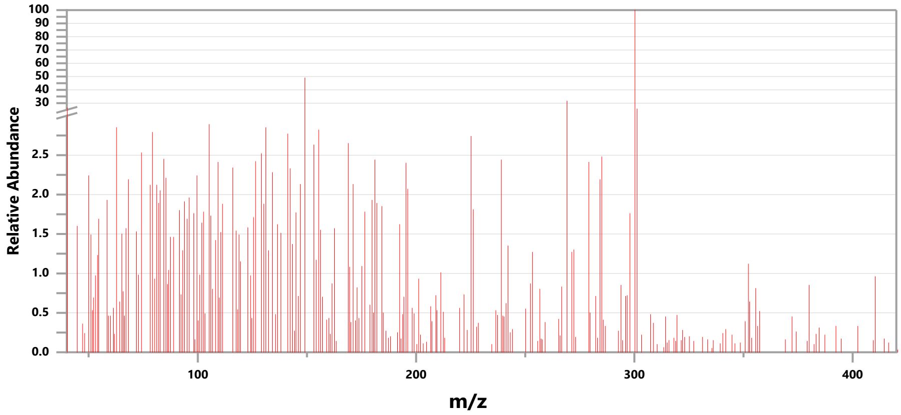

Figure S1 : Mass spectrum of the MOX-H ligand.

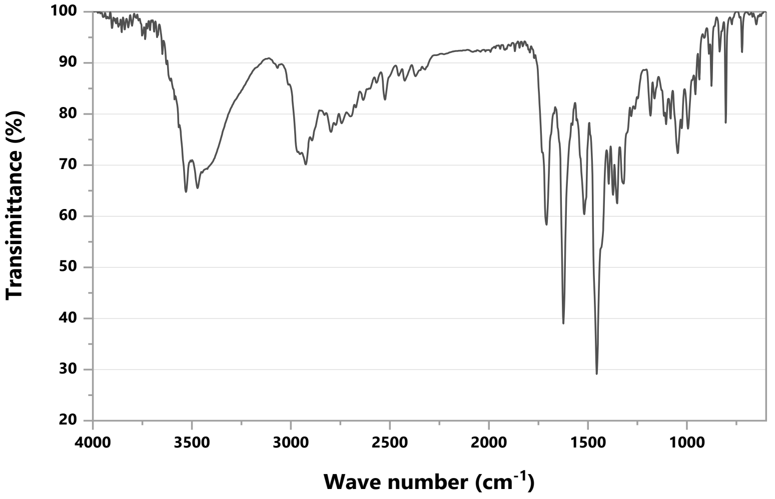

Figure S2: FT-IR spectrum of moxifloxacin hydrochloride (MF-HCl).

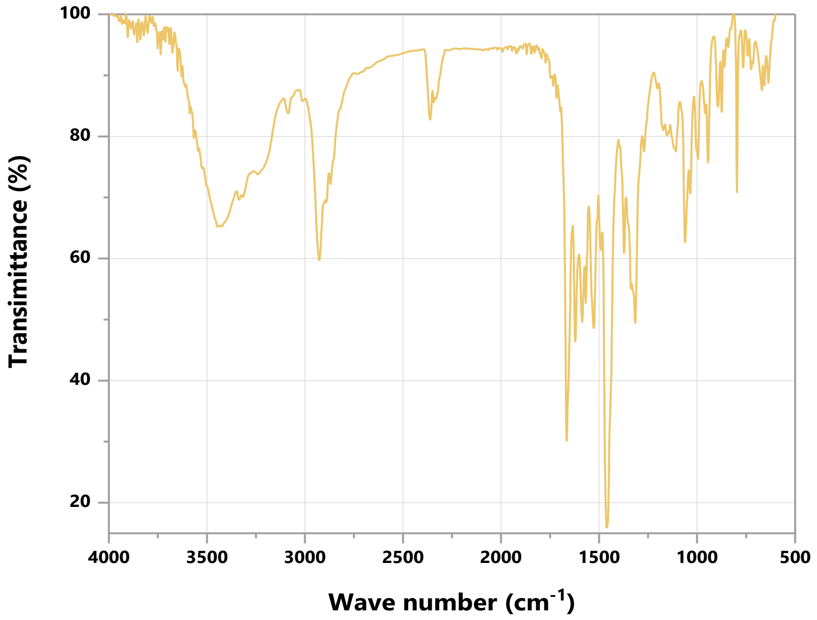

Figure S3: FT-IR spectrum of moxifloxacin hydrazide (MOX-H).

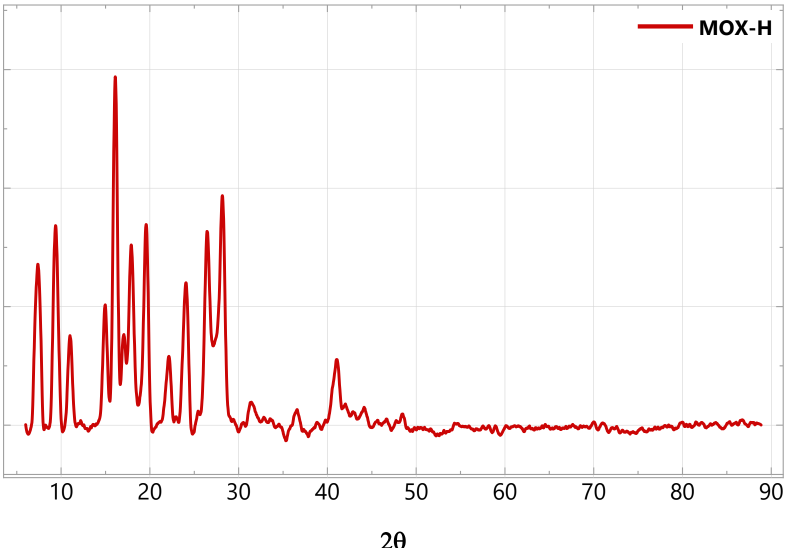

Figure S4: X-ray diffraction of MOX-F derivatives.

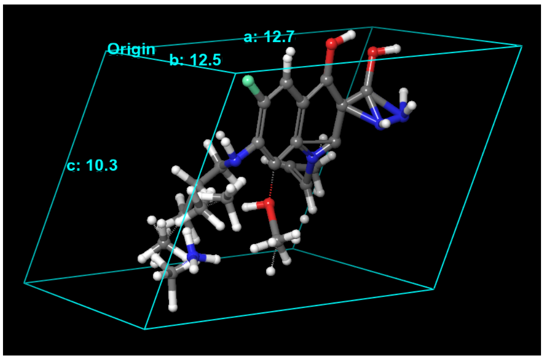

Figure S5: The unit cell view for the MOX-H.

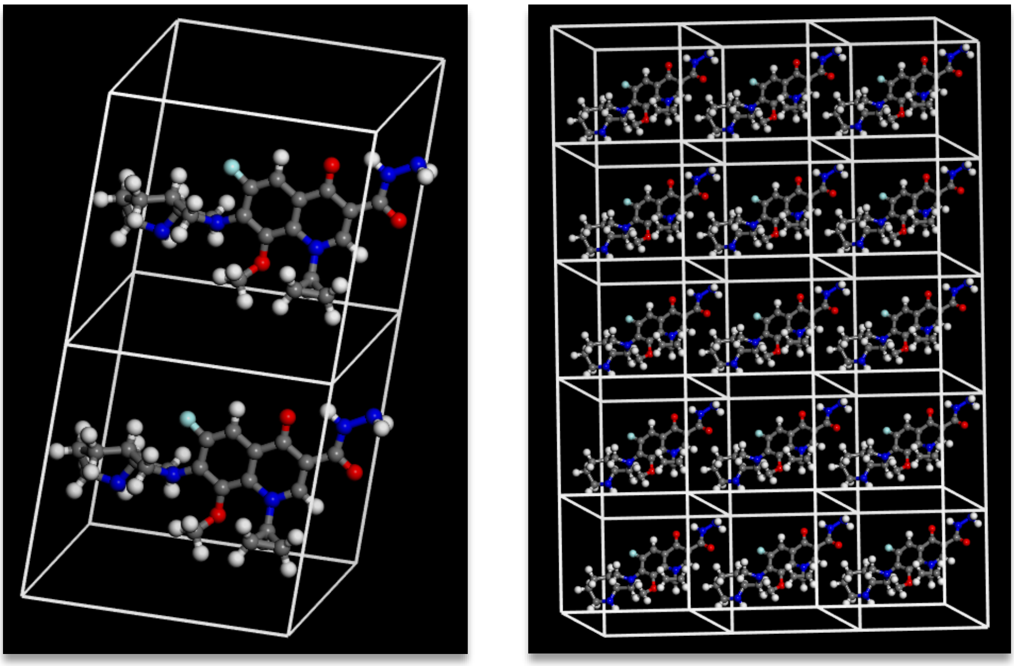

Figure S6: The package1 2 1 and 3 5 1 lattice view for the MOX-H.

Table S1. X-ray representative peaks for the MOX-H ligand.

Pos. (°2θ)	FWHM (°2θ)	d-spacing (Å)	Rel. Int. (%)	Tip width (°2θ)	ζ (nm)	δ
7.530	0.550	11.74001	51.52	0.550	**14.49**	**4.76×10^{-3}**
9.300	0.650	9.50921	63.64	0.650	**12.27**	**6.64×10^{-3}**
11.000	0.740	8.04313	43.94	0.740	**10.79**	**8.59×10^{-3}**
14.970	0.530	5.91786	53.79	0.530	**15.13**	**4.37×10^{-3}**
16.030	0.500	5.52885	100.00	0.500	**16.06**	**3.88×10^{-3}**
17.000	1.630	5.21550	50.02	1.630	**4.93**	**4.11×10^{-2}**
17.930	0.660	4.94702	68.18	0.660	**12.20**	**6.72×10^{-3}**
19.570	0.540	4.53600	74.99	0.540	**14.94**	**4.48×10^{-3}**
22.170	1.020	4.00957	48.48	1.020	**7.94**	**1.59×10^{-2}**
24.100	0.650	3.69267	63.64	0.650	**12.51**	**6.39×10^{-3}**
26.500	0.760	3.36343	72.72	0.760	**10.75**	**8.65×10^{-3}**
28.230	0.750	3.16112	74.26	0.750	**10.93**	**8.37×10^{-3}**
31.370	1.180	2.85151	26.56	1.180	**7.00**	**2.04×10^{-2}**
**Average**					**8.39**	

Table S2. The calculated crystallographic parameters for MOX-F derivatives.

**Parameters**	**MOX-H**
Molecular Formula	$C_{21}H_{26}FN_5O_3$
Molecular weight	415.466
Crystal system	Triclinic
Space group	P1
Unit cell dimension	
**a (Å)**	12.7
**b (Å)**	12.5
**c (Å)**	10.3
**α (°)**	96.1
**β (°)**	111.51
**γ (°)**	90.72
Volume (Å^3)	527.69
Volume per atom (Å^3)	50.7

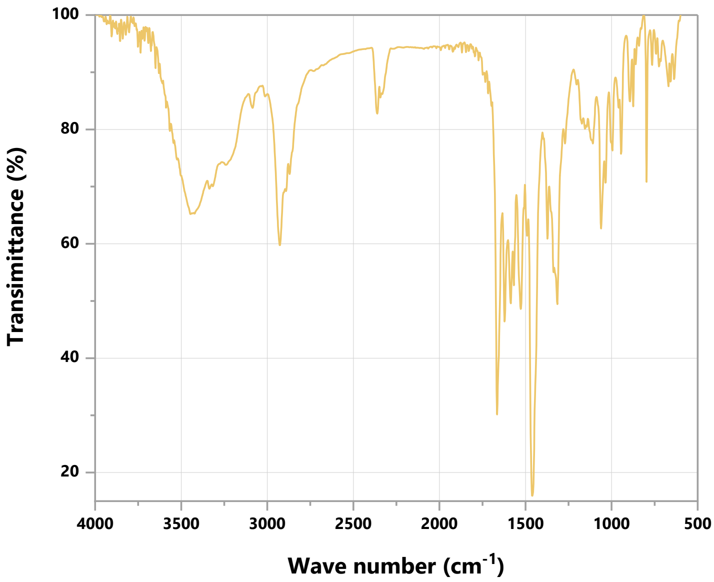

Figure S7: FT-IR spectrum of moxifloxacin hydrazide (MOX-H).

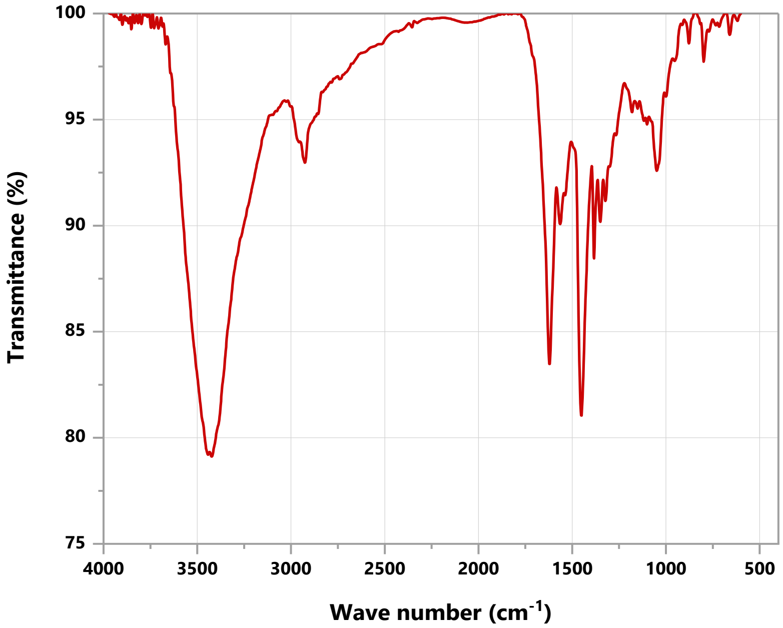

Figure S8: FT-IR spectrum of $[Co(MOX\text{-}H)_2(H_2O)Cl]Cl.2.5H_2O$.

https://doi.org/10.3390/ijms27073057

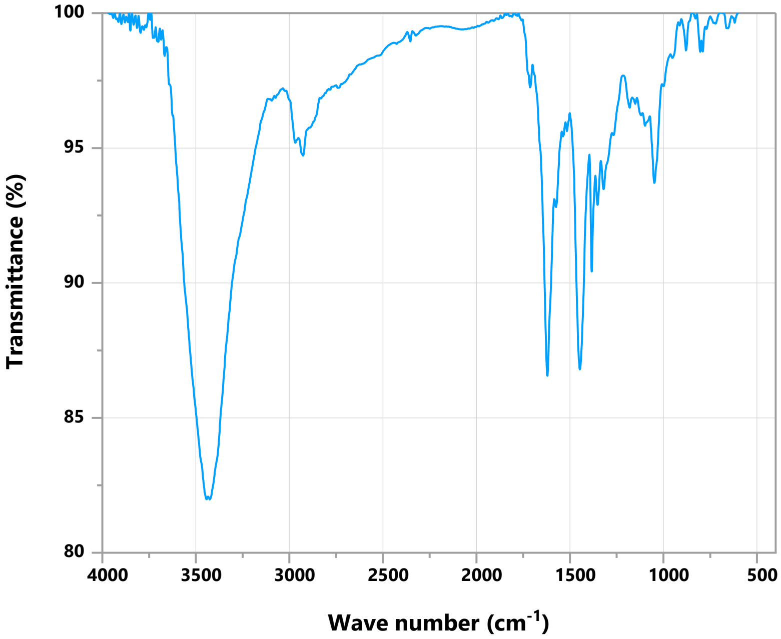

Figure S9. FT-IR spectrum of [Cu(MOX-H)$_2$(H_2O)Cl]Cl.2H_2O **(a).**

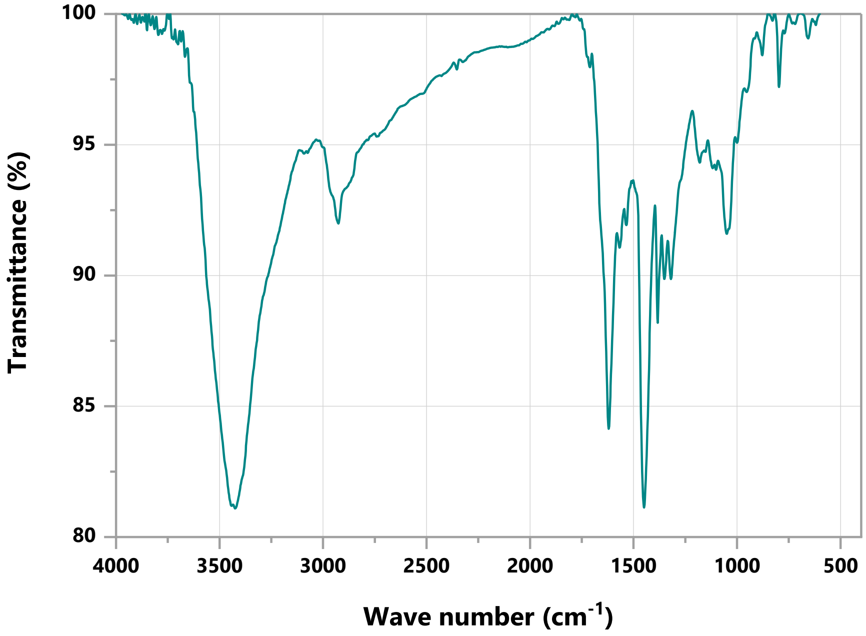

Figure S10. FT-IR spectrum of [Cu(MOX-H)$_2$(H_2O)Cl]Cl.2.5H_2O **(b)**.

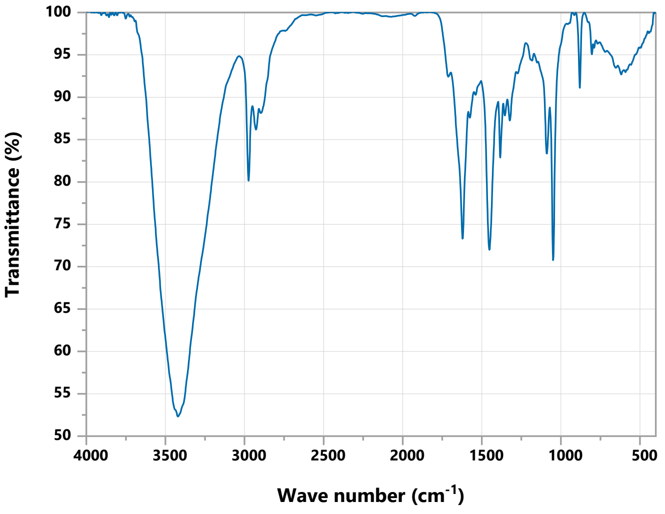

Figure S11. FT-IR spectrum of [Cu(MOX-H)$_2$(H_2O)Cl]Cl.0.5H_2O **(c)**.

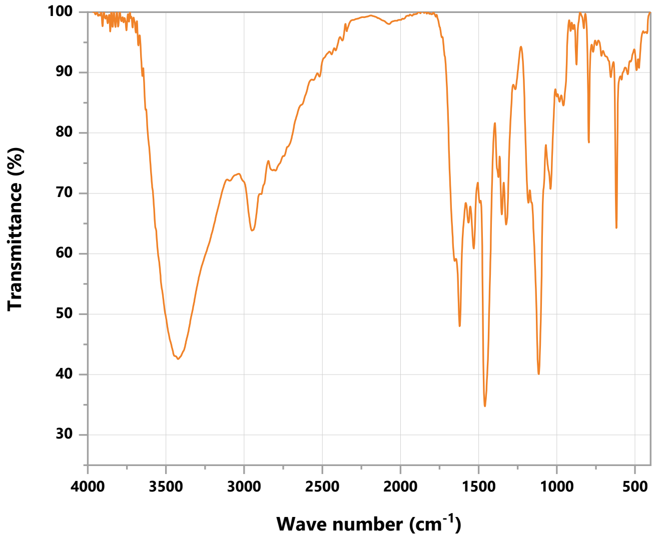

Figure S12: FT-IR spectrum of [VO(MOX-H)$_2$]SO_4.3.5H_2O.

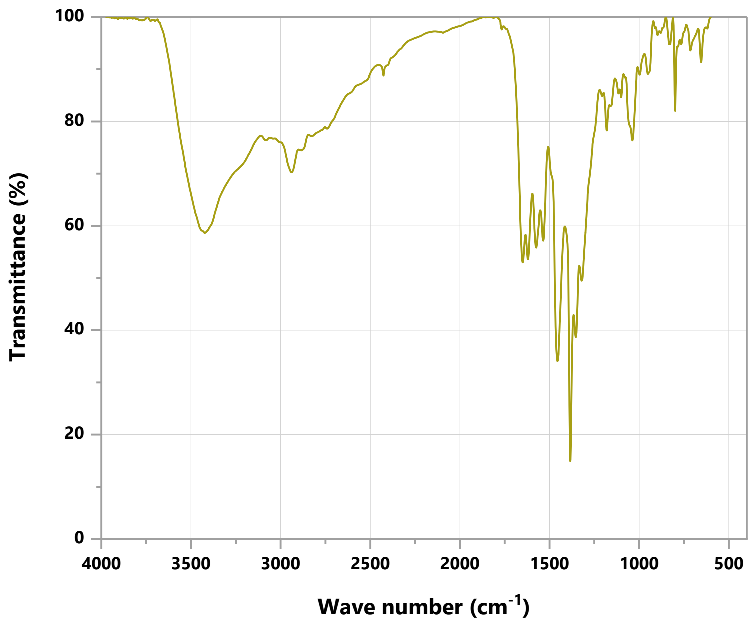

Figure S13: FT-IR spectrum of [Gd(MOX-H)$_2$(H$_2$O)(NO$_3$)$_2$]NO$_3$.2H$_2$O.

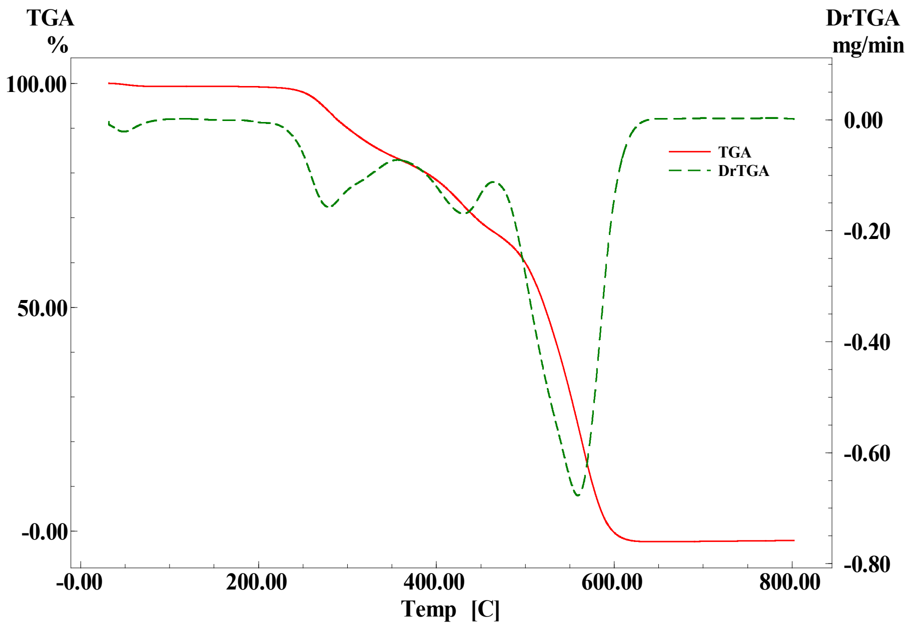

Figure S14. Thermal decomposition of (TGA and DTG) MOX-H.

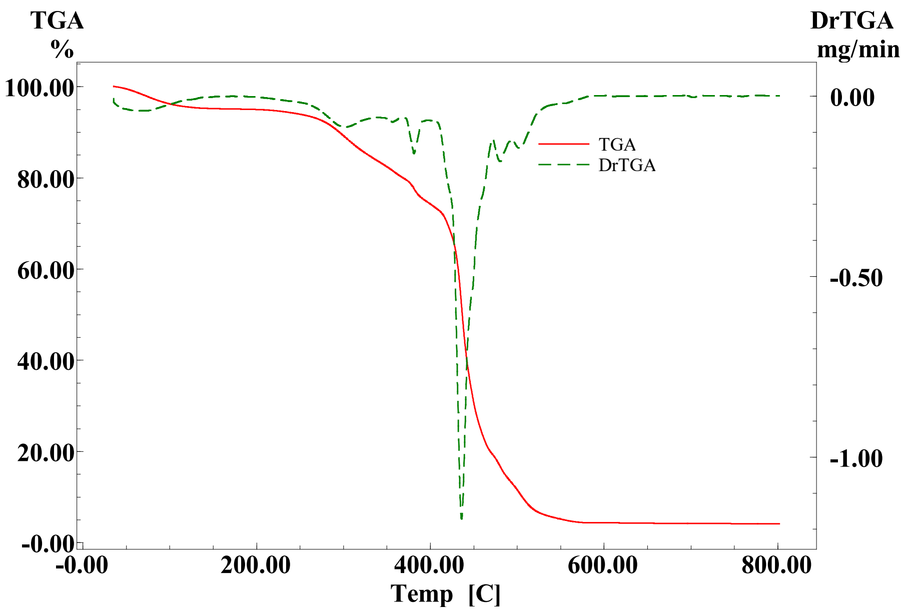

Figure S15. TGA and DTG curves of [Co(MOX-H)$_2$(H_2O)Cl]Cl.2.5H_2O.

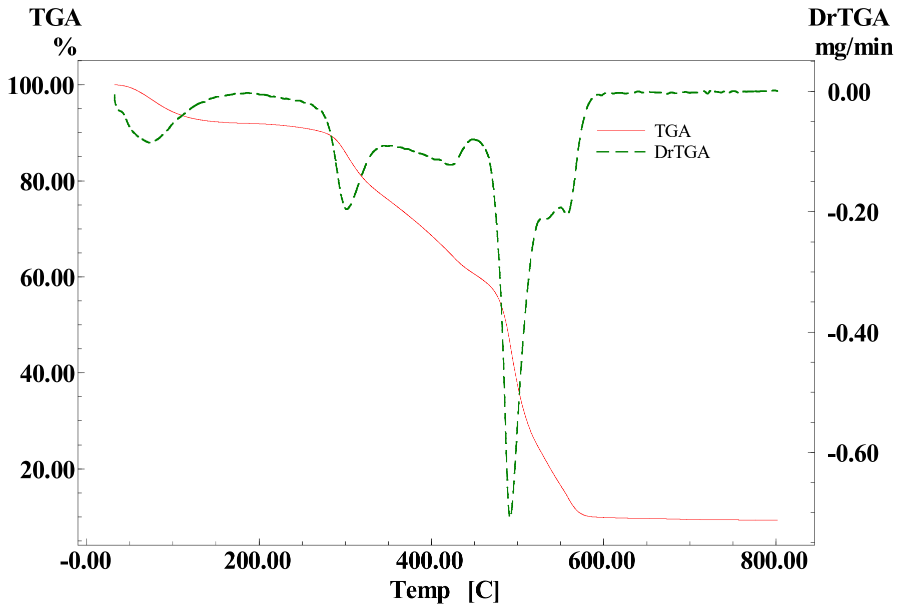

Figure S16. TGA and DTG curves of [Ni(MOX-H)$_2$(H_2O)Cl]Cl.4.5H_2O.

https://doi.org/10.3390/ijms27073057

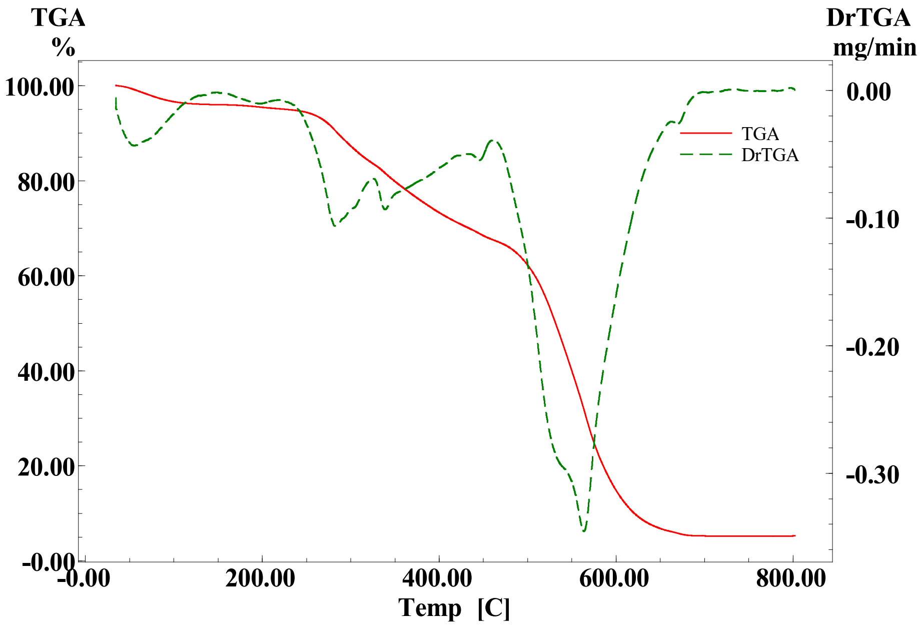

Figure S17. TGA and DTG curves of [Cu(MOX-H)$_2$(H_2O)Cl]Cl.2H_2O.

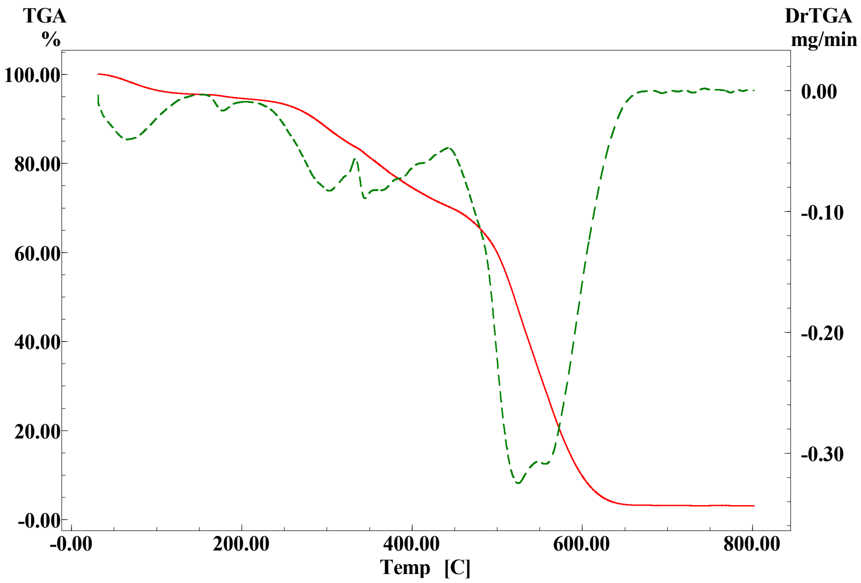

Figure S18. TGA and DTG curves of [Cu(MOX-H)$_2$(H_2O)Cl]Cl.2.5H_2O **(b)**.

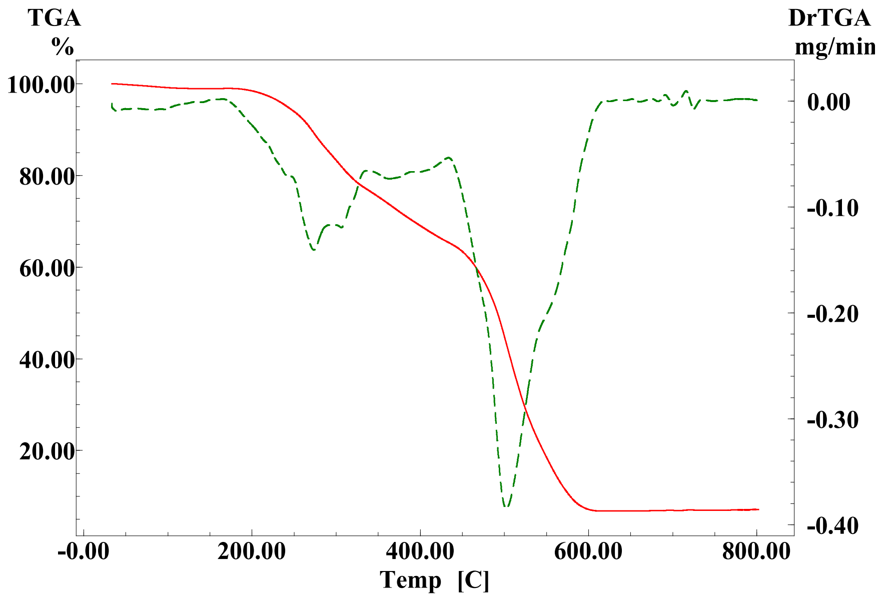

Figure S19. TGA and DTG curves of [Cu(MOX-H)$_2$(H_2O)Cl]Cl.0.5H_2O **(c)**.

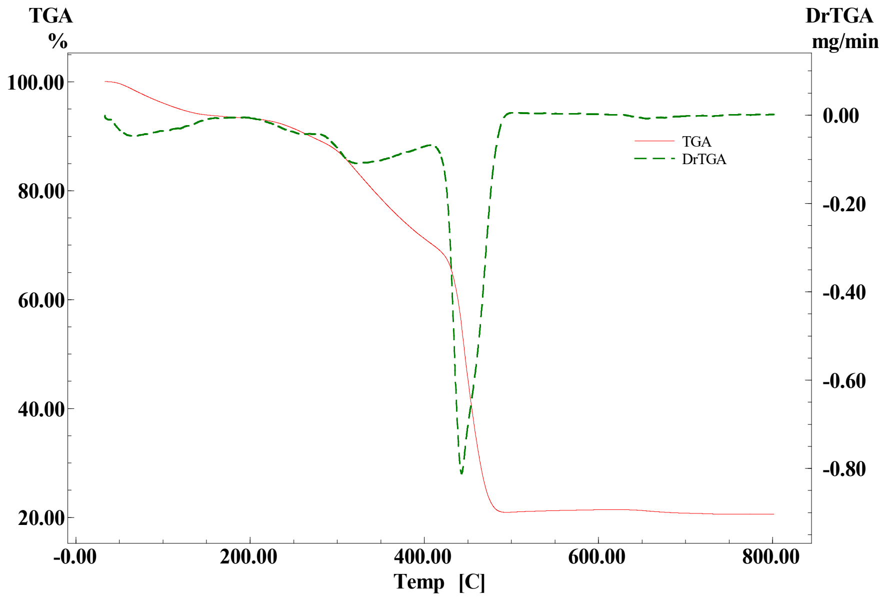

Figure S20. TGA and DTG curves of [VO(MOX-H)$_2$](SO_4) 3.5H_2O.

Table S3. TGA and DTG of MOX-H and its metal complexes.

Compound	*Temp. range °C*	*DTG temp. °C*	*Mass loss%*		*Process*	*expected products*	*Residue (Calcd.) Found*
			Found	*Calcd*			
MOX-H	31-364	278	17.4	17.4	Ligand decomposition	0.18 MOX-H	-
	364-465	432	15.4	15.4	Ligand decomposition	0.15 MOX-H	
	465-650	559	67.2	67.2	Final decomposition	0.67 MOX-H	
[Co(H_2O)Cl(MOX-H)$_2$]Cl.2.5H_2O	35-130	64	4.48	4.39	Dehydration	2.5H_2O	CoO (7.42) 5.68
	174-400	299, 380	20.8	20.25	Dechlorination + ligand decomposition	2HCl + H_2O + 0.14 MOX-H	
	406-576	436, 480	69.04	69.04	Final decomposition	0.85 MOX-H	

https://doi.org/10.3390/ijms27073057

Compound	***Temp. range °C***	***DTG temp. °C***	***Mass loss%***		***Process***	***expected products***	***Residue (Calcd.) Found***
			Found	***Calcd***			
		504					
$[Ni(H_2O)Cl(MOX\text{-}H)_2]Cl.4.5H_2O$	35-160	71	7.83	7.65	Dehydration	$4.5H_2O$	NiO+3C (6.99) 9.2
	224-450	302 422	31.15	30.54	Dechlorination + ligand decomposition	2HCl + H_2O + 0.28 MOX-H	
	451-588	492 555	51.82	50.50	Final decomposition	0.66 MOX-H	
$[Cu(H_2O)Cl(MOX\text{-}H)_2]Cl.2H_2O$ **(a)**	34-127	56	3.80	3.50	Dehydration	$2H_2O$	CuO (7.8) 5.35
	146-462	283 338 446	28.57	27.67	Coordination sphere + ligand decomposition	2HCl + H_2O + 0.23 MOX-H	
	462-692	562	62.10	61.13	Final decomposition	0.75 MOX-H	
$[Cu(H_2O)Cl(MOX\text{-}H)_2]Cl.2.5H_2O$ **(b)**	31-150	68	4.57	4.37	Dehydration	$2.5H_2O$	Cu (6.17) 3.23
	150-445	176 304 347	25.19	25.00	Coordination sphere + ligand decomposition	2HCl + H_2O + 0.20 MOX-H	
	445-660	521 559	67.00	66.25	Final decomposition	0.82 MOX-H	
$[Cu(H_2O)Cl(MOX\text{-}H)_2]Cl.0.5H_2O$ **(c)**	33-167	72	1.06	0.906	Dehydration	$0.5H_2O$	CuO (6.39) 6.68
	167-436	273 306	33.94	32.60	Coordination sphere + ligand decomposition	2HCl + H_2O + 0.28 MOX-H	
	436-616	521 559	58.36	58.61	Final decomposition	0.70 MOX-H	
$[VO(MOX\text{-}H)_2(SO_4)]3.5H_2O$	34-165	61	6.17	5.96	Dehydration	$3.5H_2O$	V_2O_3+7C (21.9) 20.99
	200-411	260 315	23.44	23.35	Sulfate removal + ligand decomposition	SO_2+ 0.22 MOX-H	
	411-501	422	48.86	48.74	Final decomposition	0.62 MOX-H	
$[Gd(H_2O)(MOX\text{-}H)_2(NO_3)_2]NO_3.2H_2O$	37-145	62	2.87	3.08	Dehydration	$2H_2O$	Gd+6C (18.6) 17.5
	181-462	213 283 432	38.89	39.27	Coordination sphere (NO_3 liberation + H_2O) + ligand decomposition	$3HNO_3$ + H_2O + 0.31 MOX-H	
	462-628	499 588	40.69	40.59	Final decomposition	+ 0.60 MOX-H	

Table S4. The kinetic parameters for selected decomposition steps of the MOX-H and its complexes.

Compound	***Step***	R^2	***Order* (n)**	**T (K)**	**ΔE_a (J/mol)**	**Z**	**ΔS^* (J/K.mol)**	**ΔH^* (KJ/mol)**	**ΔG^* (KJ/mol)***
MOX-H	Ligand decomposition	0.97	2	833	211.16	1.11×10^{13}	-3.7117	-6.72	-3.62
Co-MOXH	Ligand decomposition	0.98	2	709	510.48	3.24×10^{37}	466.06	-5.39	-335.89
Ni MOXH	Ligand decomposition	0.96	2	764	368.01	6.15×10^{24}	221.85	-5.99	-175.51
Cu-MOXH	Ligand decomposition	0.99	2	835	119.99	1.04×10^{7}	-119.12	-6.82	92.67
VO-MOXH	Ligand decomposition	0.98	2	695	266.21	1.00×10^{19}	111.81	-5.51	-83.24
Gd-MOXH	Ligand decomposition	0.99	2	770	234.70	1.63×10^{15}	38.41	-6.17	-35.76

https://doi.org/10.3390/ijms27073057

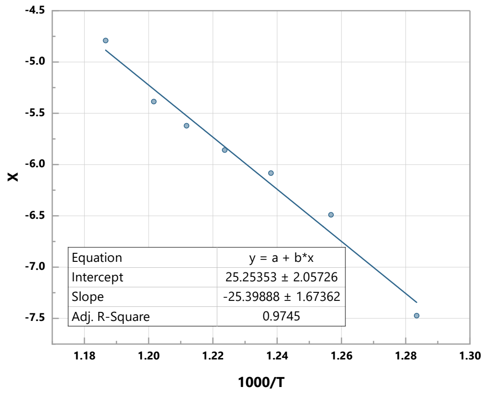

Fit-linear curve of ligand decomposition of **MOX-H .**

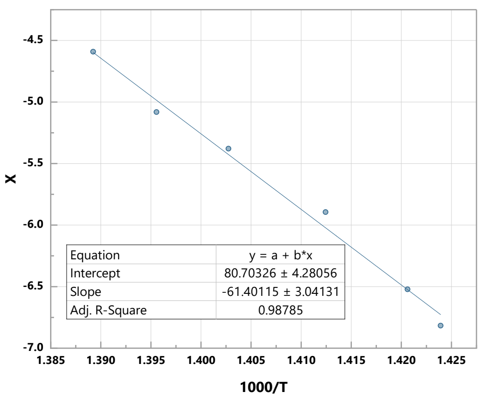

Fit-linear curve of coordination sphere decomposition of **Co-MOXH** complex.

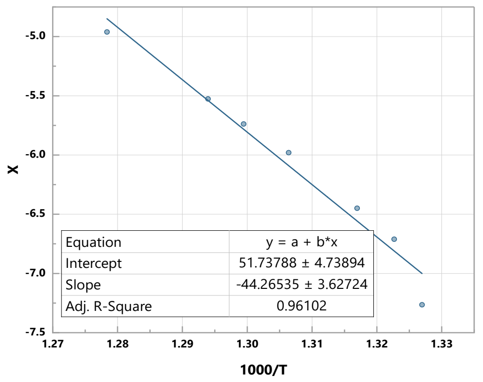

Fit-linear curve of coordination sphere decomposition of **Ni-MOXH** complex.

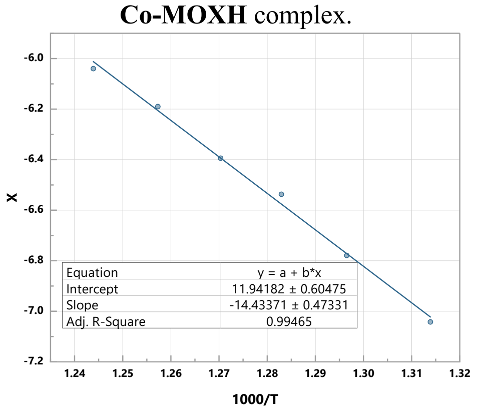

Fit-linear curve of coordination sphere decomposition of **Cu-MOXH** complex.

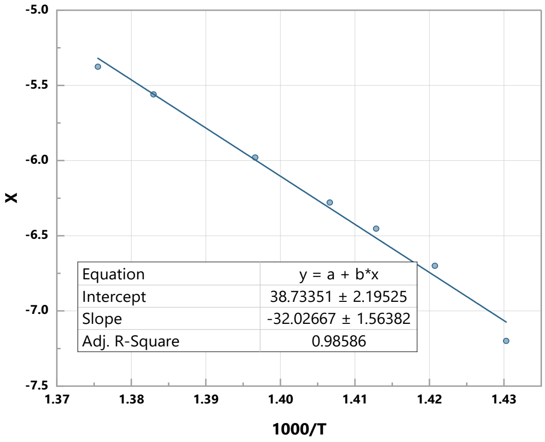

Fit-linear curve of coordination sphere decomposition of **VO-MOXH** complex.

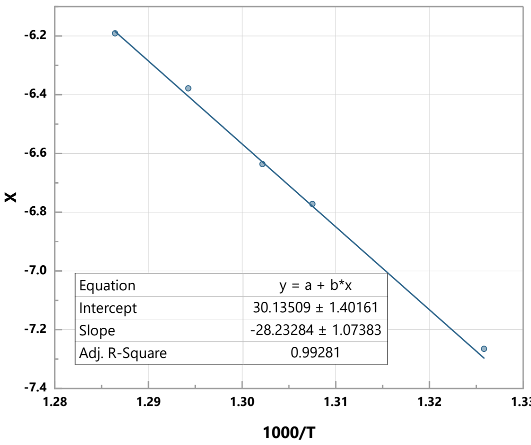

Fit-linear curve of coordination sphere decomposition of **Gd-MOXH** complex.

Figure S21. Fit-linear curve of ligand decomposition steps of MOX-H and its complexes. **X** = ln[-ln(1-α)/T^2] for n ± 1 or ln[1-(1-α)$^{1-n}$ / (1-n)T^2] for n=1.

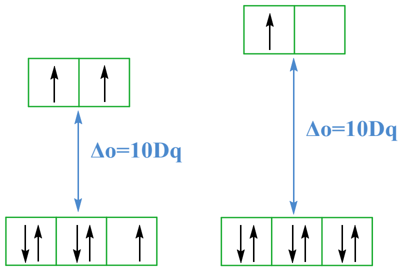

high spin

S = 3×1/2 = 3/2

2S + 1 = 4

low spin

S = 1×1/2 = 1/2

2S + 1 = 2

Figure S22: d-orbitals in the O_h ligand field for the electron configuration d^7

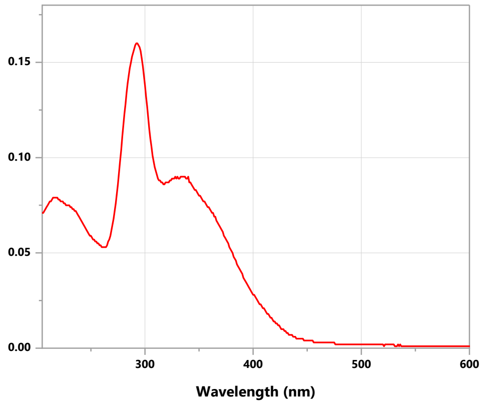

Figure S23: UV-Vis spectrum of the Co-MOX-H complex.

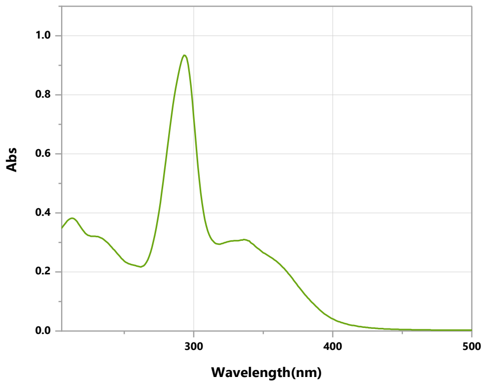

Figure S24. UV-Vis spectrum of the Ni-MOX-H complex.

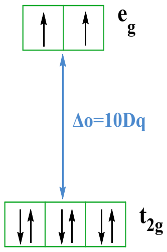

S=2×1/2 =1

2S+1=3

Figure S25. d-orbitals in the O_h ligand field for the electron configuration d^8.

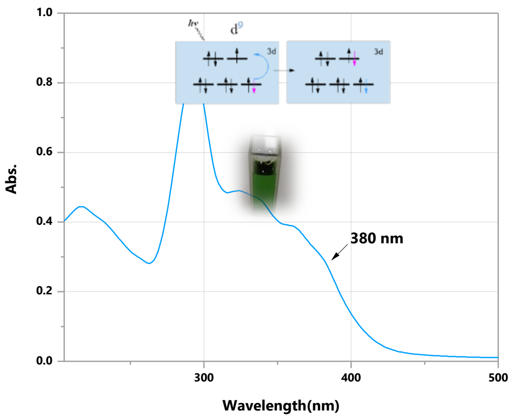

Figure S26. UV-visible spectrum of a **Cu(II)-MOX-H (a)** complex.

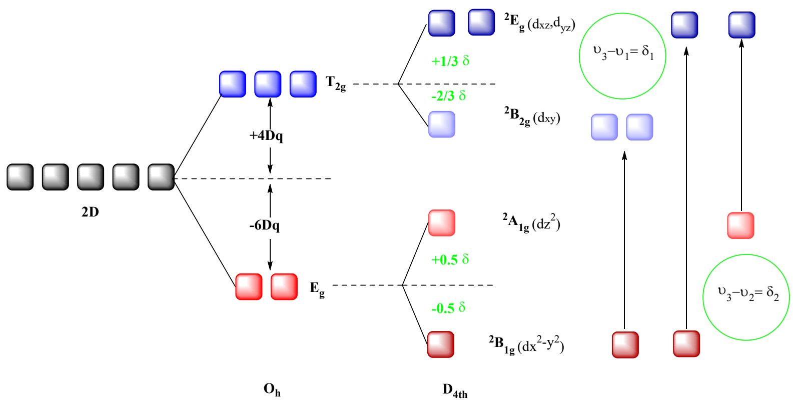

Figure S27. The d-d transition of Cu(II) (d[9]configuration).

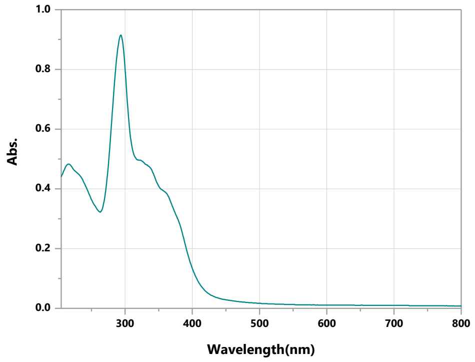

Figure S28. UV-visible spectrum of **Cu(II)-MOX-H (b)** complex.

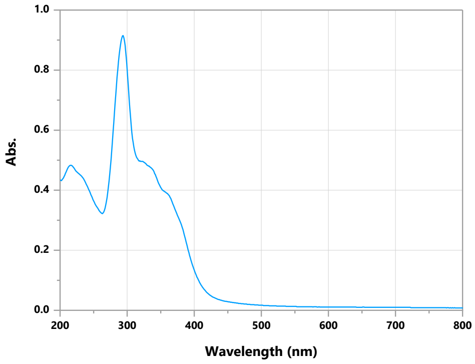

Figure S29. UV-visible spectrum of **Cu(II)-MOX-H (c)** complex.

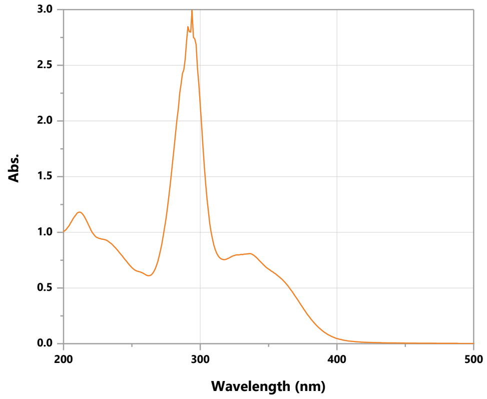

Figure S30. UV-Vis spectrum of the VO-MOX-H complex.

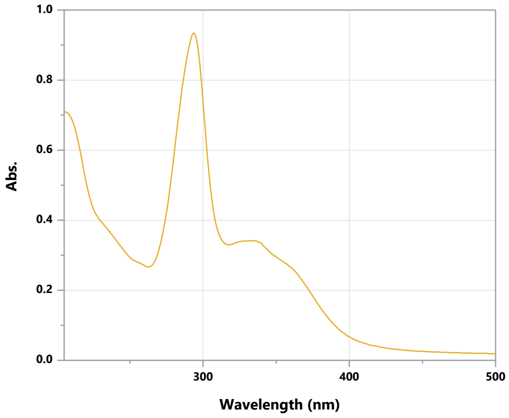

Figure S31. UV-Vis spectrum of the Gd-MOX-H complex.

Table S5. ESR spectral parameters of Cu(II) and VO(IV) complexes.

Complex	g‖	g⊥	g_{av}	G	K	A‖ ($\times 10^{-4}$ cm^{-1})	A⊥ ($\times 10^{-4}$ cm^{-1})	A(iso) ($\times 10^{-4}$ cm^{-1})	Bonding parameter
**Cu(II)**	2.26	2.03	2.11	11.0	0.61	190	-	-	$\alpha^2 = 0.84$
**VO(IV)**	1.96	1.98	1.97	1.9	0.92	167	60.6	96.1	k = 0.92

Table S6. The magnetic properties and electronic spectra of MOX-H ligand and its complexes.

**Compound**	**λ_{max}**		**Assignment**	**10Dq**		**CFSE***	**μ_{eff}**	**Postulated Geometry**
	**(nm)**	**(cm^{-1})**		**(cm-1)**	**(kJ/mol)**		**(BM)**	
**MOX-H**	358	27933	n→π*					
	336	29762						
	294	33898	π→π*					
	230	43478						
	211	47393						
**[Co(H_2O)Cl(MOX-H)$_2$]Cl.2.5H_2O**	**500**	**20000**	**$^4T_{1g}(F)\rightarrow{}^4T_{1g}(P)$**				**2.92**	**Distorted Octahedral**
	360	27778	n→π*					
	335	29851						
	291	34364	π→π*					
	233	42918						
	212	47170						
**[Ni(H_2O)Cl(MOX-H)$_2$]Cl.4.5H_2O**	**599**	**16694**	**$^3A_{2g}(F)\rightarrow{}^3T_{1g}(F)$**	**9274**	**111.79**	**-133 + 3δ**	**2.1**	**Distorted Octahedral**
	**553**	**18083**	**$^3A_{2g}(F)\rightarrow{}^3T_{1g}(P)$**					
	360	27778	n→π*					
	336	29762						
	293	34130	π→π*					
	231	43290						
**[Cu(H_2O)Cl(MOX-H)$_2$]Cl.2H_2O**	**658**	**15197**	**$^2B_{1g}\rightarrow{}^2B_{2g}$**	**15197**	**184**	**-110 + 4δ**	**0.6**	**Distorted Octahedral**
	**380**	**26315**	**$^2B_{1g}\rightarrow{}^2E_g$**					
	360	27778	n→π*					
	323	30960						

https://doi.org/10.3390/ijms27073057

Compound	λ_{max} (nm)	λ_{max} (cm^{-1})	Assignment	10Dq (cm-1)	10Dq (kJ/mol)	CFSE*	μ_{eff} (BM)	Postulated Geometry
	293	34130	π→π*					
	216	46296						
**[VO(MOX-H)$_2$(SO$_4$)]. 3.5H$_2$O**	355	28169	LMCT				**0.43**	**Distorted Octahedral**
	337	29674	n→π*					
	291	34364	π→π*					
	230	43478						
	212	47170						
**[Gd(H$_2$O)(NO$_3$)$_2$(MOX-H)$_2$]NO$_3$.2H$_2$O**	361	27701	n→π*				**5.98**	**Pentagonal Bipyramidal**
	336	29762						
	294	34014	π→π*					
	236	42373						
	212	47170						

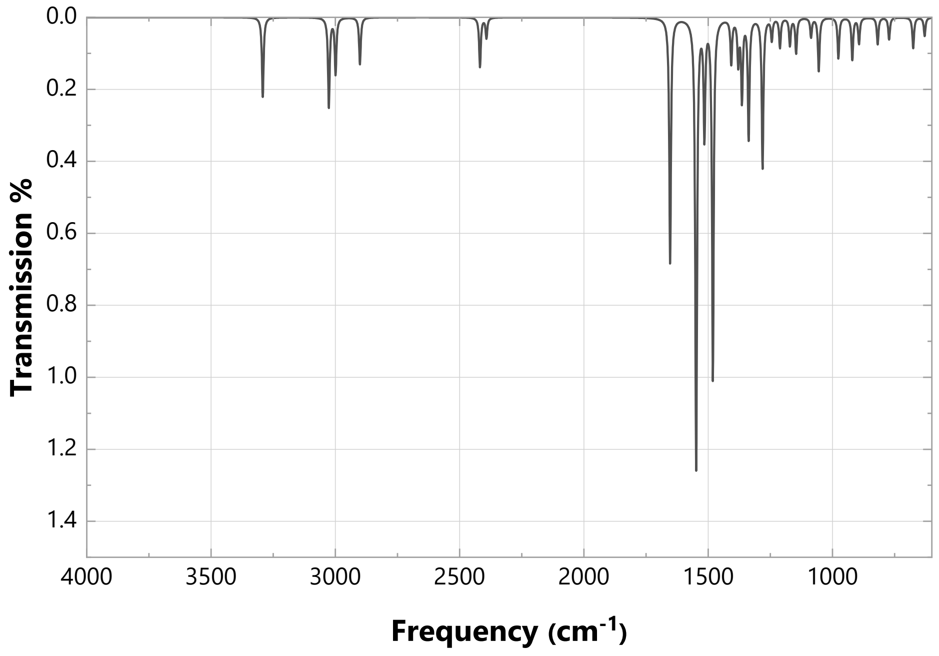

Figure S32. Calculated FT-IR spectral data of MOX-H.

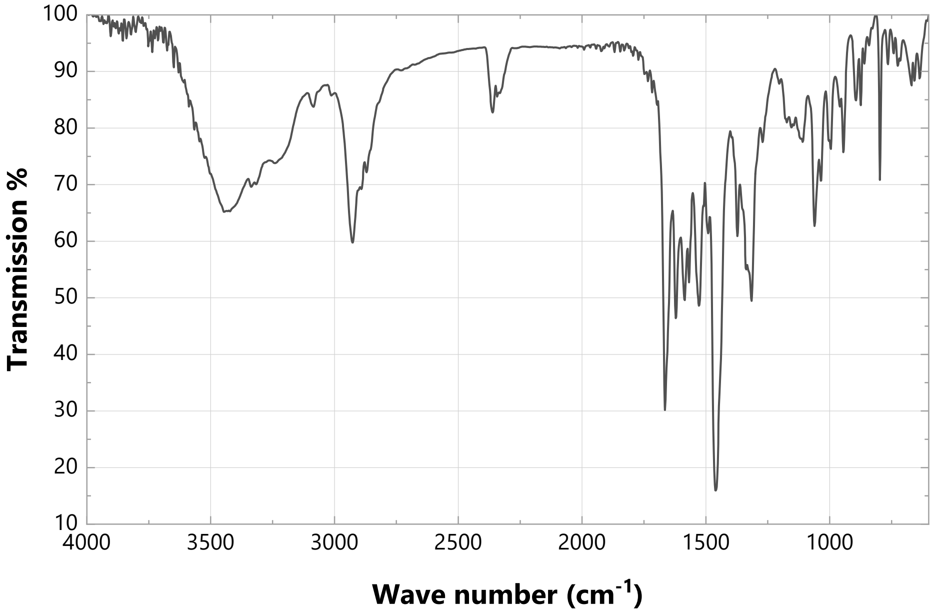

Figure S33. Experimental FT-IR spectrum of MOX-H.

Table S7. Comparative toxicity probabilities of MOX-F and MOX-H (ProTox 30).

Target	**Compounds**	
	**MOX-F**	**MOX-H**
Hepatotoxicity	**0.57 (Inactive)**	0.52 (Active)
Neurotoxicity	**0.95 (Active)**	0.82 (Active)
Nephrotoxicity	**0.91 (Active)**	0.58 (Active)
Respiratory toxicity	**0.93 (Active)**	0.88 (Active)
Clinical toxicity	**0.86 (Active)**	0.73 (Active)
Immunotoxicity	0.98 (Inactive)	**0.95 (Active)**
Mutagenicity	0.52 (Active)	0.56 (Active)
Cardiotoxicity	0.80 (Inactive)	0.78 (Inactive)
BBB Penetration	0.76 (Inactive)	0.63 (Inactive)

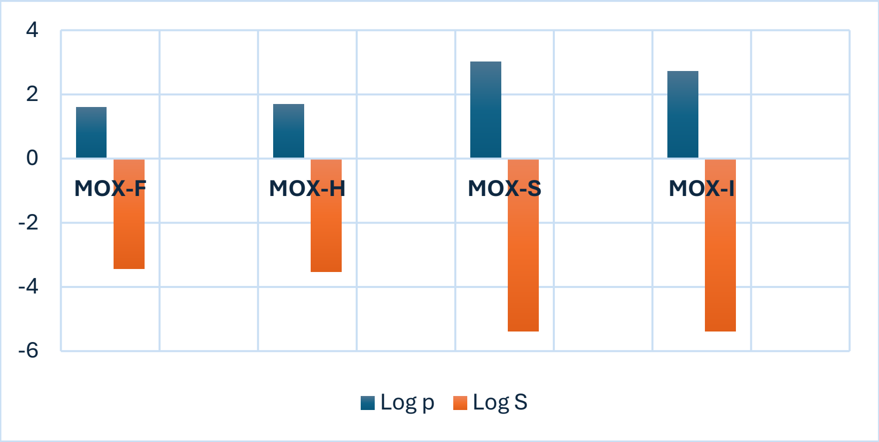

Figure S34. Column Chart comparing LogP and LogS values of MOX-F derivatives.

Table S8. Molecular properties, pharmacokinetics and toxicity and of MOX-F and its analogous.

<table>
<tr><th colspan="2" rowspan="2">Property</th><th colspan="4">Predicted Values</th></tr>
<tr><th colspan="2">MOX-F</th><th colspan="2">MOX-H</th></tr>
<tr><td rowspan="11">Molecular properties</td><td>M.wt</td><td colspan="2">437.89</td><td colspan="2">415.20</td></tr>
<tr><td>Lipophilicity (LogP)</td><td colspan="2">1.60</td><td colspan="2">1.69</td></tr>
<tr><td>Solubility (LogS)</td><td colspan="2">-3.43</td><td colspan="2">-3.52</td></tr>
<tr><td rowspan="3">Topological Polar Surface Area (TPSA)</td><td colspan="2">83.80</td><td colspan="2">101.62</td></tr>
<tr><td>HBD</td><td>HBA</td><td>HBD</td><td>HBA</td></tr>
<tr><td>2</td><td>6</td><td>3</td><td>6</td></tr>
<tr><td>NRBs</td><td colspan="2">4</td><td colspan="2">5</td></tr>
<tr><td>Molar Refractivity</td><td colspan="2">121.01</td><td colspan="2">117.98</td></tr>
<tr><td>pk$_a$ (Basic/Acidic)</td><td colspan="2">8.59/5.52</td><td colspan="2">8.59/12.64</td></tr>
<tr><td>Bioavailability Score</td><td colspan="2">0.55</td><td colspan="2">0.55</td></tr>
<tr><td>Drug Likeness Score</td><td colspan="2">0.71</td><td colspan="2">0.92</td></tr>
<tr><td rowspan="6">Absorption</td><td>Caco2 Permeability</td><td colspan="2">1.137</td><td colspan="2">1.004</td></tr>
<tr><td>Intestinal Absorption</td><td colspan="2">93.264</td><td colspan="2">84.219</td></tr>
<tr><td>Skin Permeability</td><td colspan="2">-2.735</td><td colspan="2">-2.915</td></tr>
<tr><td>P-Gp Substrate</td><td colspan="2">Yes</td><td colspan="2">Yes</td></tr>
<tr><td>P-Gp I Inhibitor</td><td colspan="2">No</td><td colspan="2">No</td></tr>
<tr><td>P-Gp II Inhibitor</td><td colspan="2">No</td><td colspan="2">No</td></tr>
<tr><td rowspan="2">Dist</td><td>BBB Permeability</td><td colspan="2">-1.021</td><td colspan="2">-0.742</td></tr>
<tr><td>CNS Permeability</td><td colspan="2">-2.649</td><td colspan="2">-3.55</td></tr>
<tr><td rowspan="6">Toxicity</td><td>AMES Toxicity</td><td colspan="2">No</td><td colspan="2">No</td></tr>
<tr><td>Max. Tolerated Dose</td><td colspan="2">0.258</td><td colspan="2">-0.417</td></tr>
<tr><td>Acute Toxicity (LD50)</td><td colspan="2">2.568</td><td colspan="2">3.011</td></tr>
<tr><td>Chronic Toxicity</td><td colspan="2">1.271</td><td colspan="2">1.729</td></tr>
<tr><td>T.Pyriformis Toxicity</td><td colspan="2">0.289</td><td colspan="2">0.289</td></tr>
<tr><td>Minnow Toxicity</td><td colspan="2">0.996</td><td colspan="2">2.635</td></tr>
</table>

* **HD**: Hydrogen donor, **HA**: Hydrogen acceptor, **P-gp** :P-glycoprotein, **NRBs:** Number of rotatable bonds.

https://doi.org/10.3390/ijms27073057

.

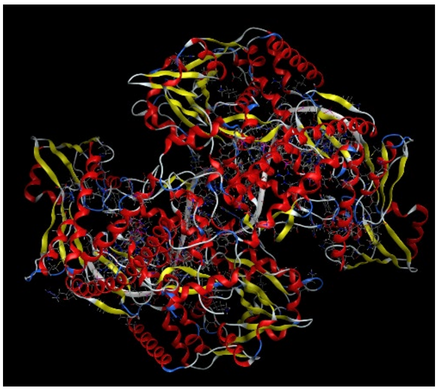

Figure S35. Crystal structure of human topoisomerase II alpha in complex with DNA and etoposide (PDB ID: 5GWK).

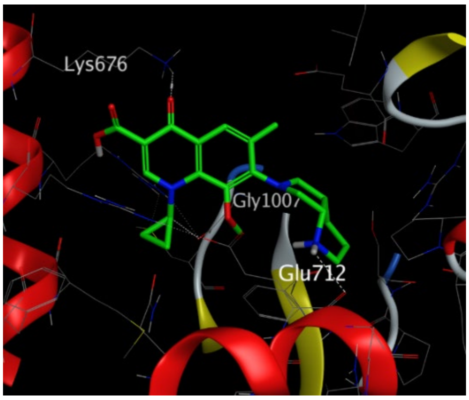

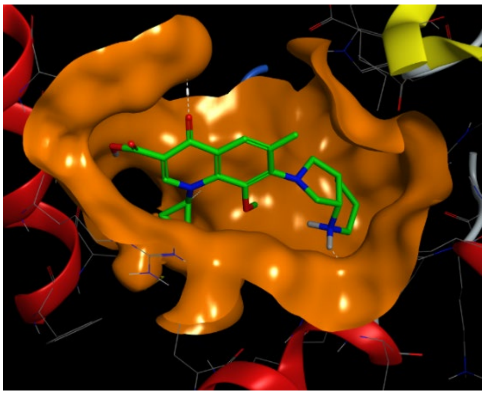

(A) **(B)**

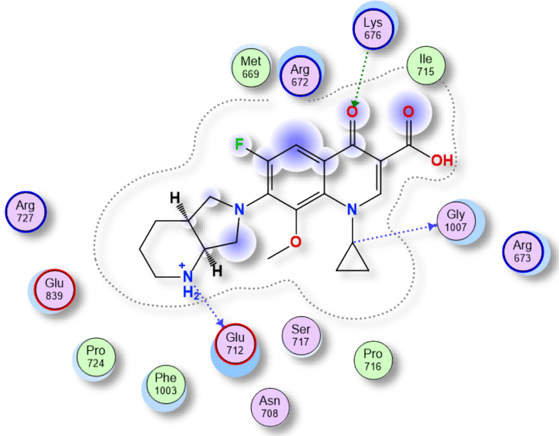

(C)

Figure S36. Docking model of the interaction of MOX-F with Topoisomerase II (PDB code: 5GWK): **(A)** 3D interaction diagram, **(B)** Cavity form **and (C)** 2D interaction diagram.

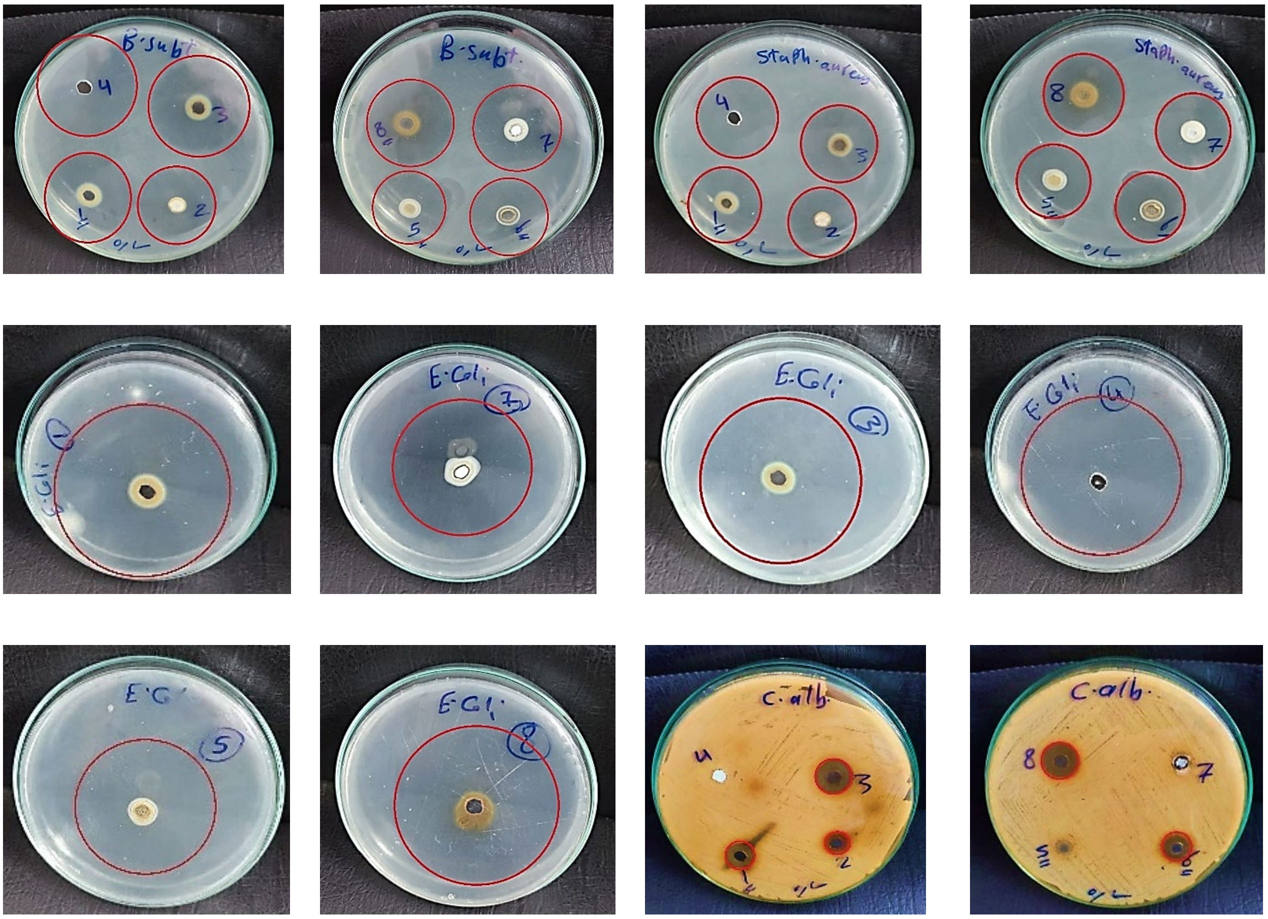

Figure S37. Representative agar plates showing inhibition zones of MOX-H and its metal complexes against *S. aureus*, *B. subtilis*, *E. coli*, and *C. albicans*. Metal complexes (notably Cu, Co, Ni) display larger clear zones than the free ligand.

Table S9: The inhibition zone (mm) of the **MOX-H** ligand and their complexes towards some Gram-positive, Gram-negative bacteria [illegible] fungi.

Tested Microorganisms	Sample Code	MOX-F	MOX-H	Co-MOXH	Ni-MOXH	Cu-MOXH (a)	Cu-MOXH (b)	Cu-MOXH (c).	VO- MOXH	Gd-MOXH	Control Drug
**Gram Positive Bacteria**											**Gentamycin**
Staphylococcus aureus	ATCC 25923	**53**	43	**48**	44	49	37	**51**	43	41	24
Bacillus subtilis	RCMB 015 (1) NRRL B-543	**48**	42	**47**	39	44	35	**47**	42	45	26
**Gram Negative Bacteria**											**Gentamycin**
Escherichia coli	ATCC 25922	65	62	**71**	58	**75**	23	59	50	63	30
**Fungi**											**Ketoconazole**

Candida albicans	RCMB 005003 (1) ATCC 10231	**NA**	12	**18**	16	13	16	14	10	12	20

Table S10: MIC of the MOX-H ligand and their complexes towards some Gram-positive, Gram-negative bacteria and fungi (µM).

**Tested microorganisms**	**Sample code**	**MOX-F**	**MOX-H**	**Co-MOXH**	**Ni-MOXH**	**Cu-MOXH**	**VO-MOXH**	**Gd-MOXH**
**Gram Positive Bacteria**								
Staphylococcus aureus	ATCC 25923	**4.88**	19.53	**4.88**	9.76	9.76	**4.88**	19.53
	µM	**11.2**	**47.1**	4.8	**9.2**	**9.6**	4.6	15.9
Bacillus subtilis	RCMB 015 (1) NRRL B-543	**4.88**	9.76	9.76	**4.88**	**4.88**	19.53	9.76
	µM	**11.2**	23.5	9.5	**4.6**	**4.8**	18.4	7.95
**Gram Negative Bacteria**								
Escherichia coli	ATCC 25922	39.06	1250	**78.13**	312.5	78.13	312.5	625
	µM	89.4	3008	**76.3**	294.9	76.6	295.7	508.9
**Fungi**								
Candida albicans	RCMB 005003 (1) ATCC 10231	NA	5000	**625**	**625**	5000	5000	1250
	µM	NA	12034	**610**	**589**	4904	4730	1017

https://doi.org/10.3390/ijms27073057

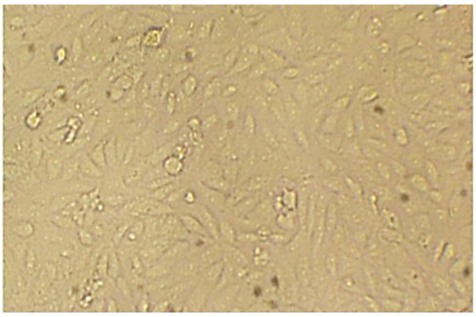

Figure S38: Control MCF-7 *(breast cancer)* cell line.

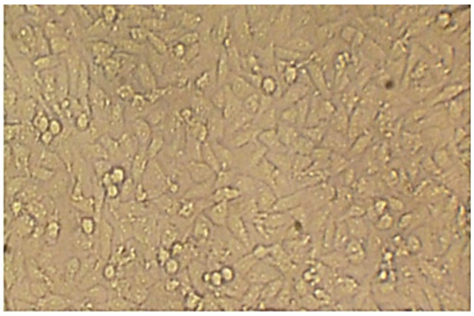

Figure S39: Control HepG2 *(liver cancer)* cell line.

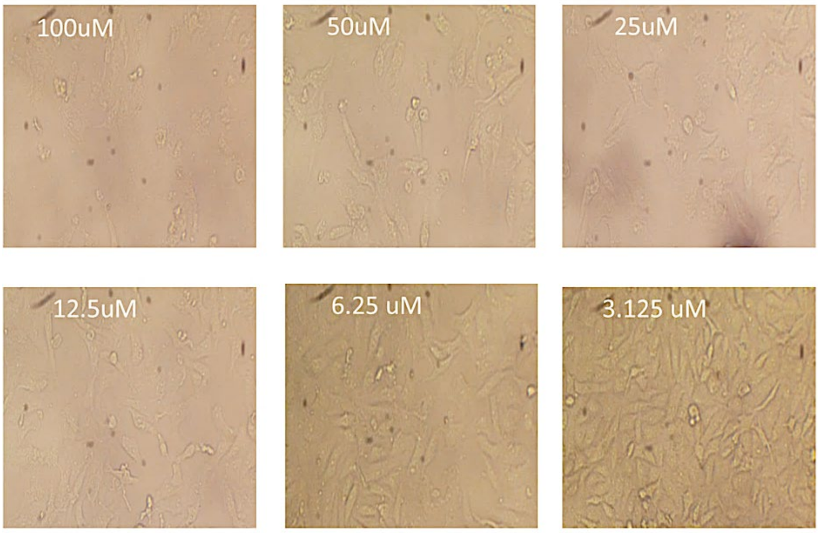

Figure S40: Effect of MOX-H on MCF-7 cells at different concentrations.

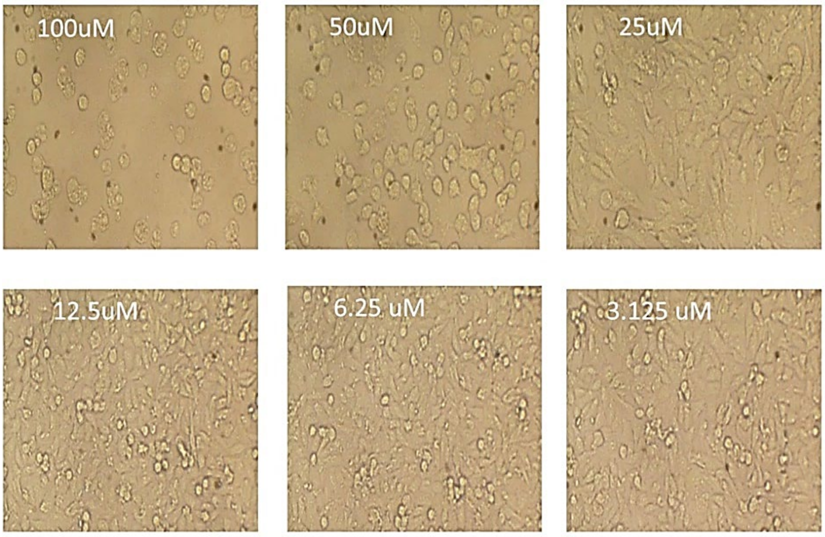

Figure S41: Effect of MOX-H on HepG2 cells at different concentrations.

https://doi.org/10.3390/ijms27073057

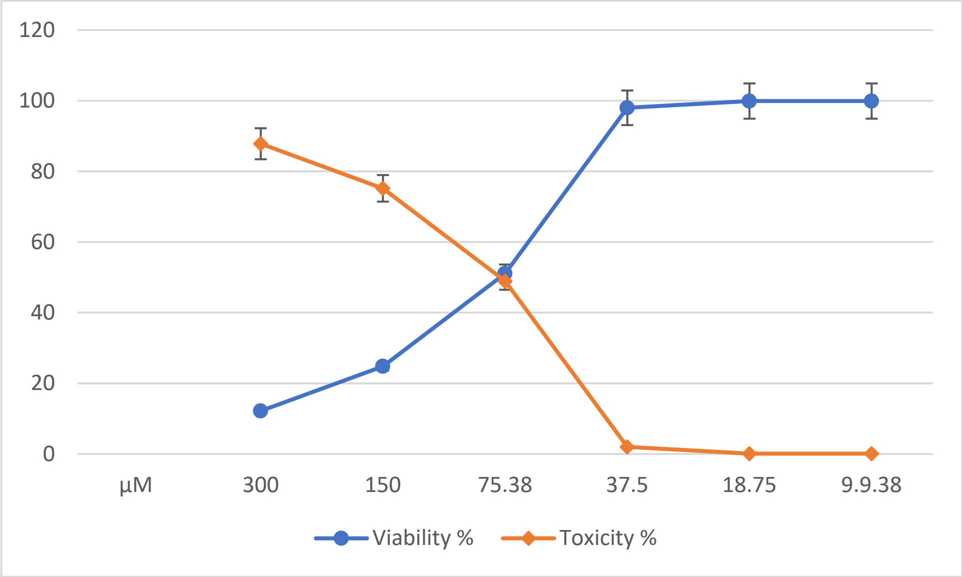

Figure **S42**: Effect of MOX-F on Vero cells at different concentrations

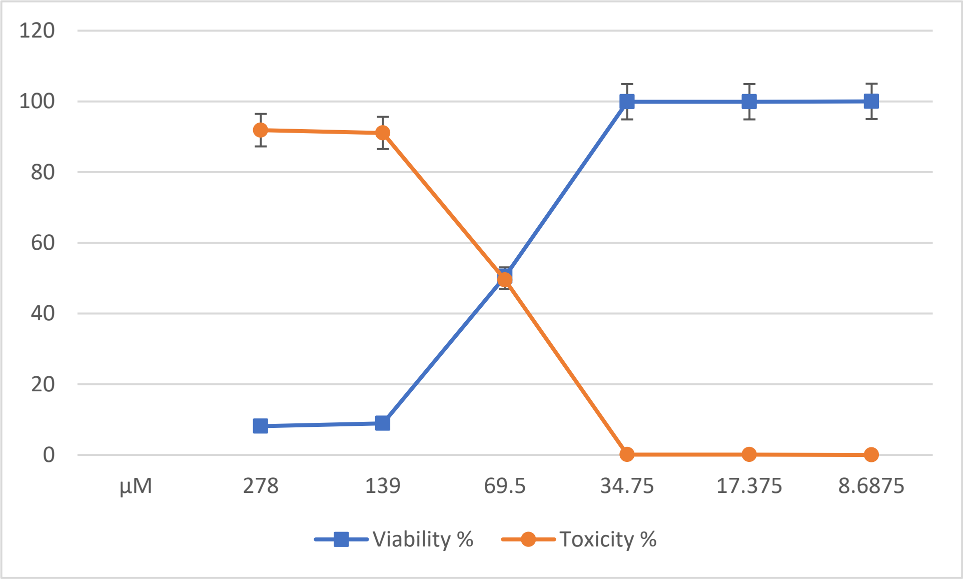

Figure **S43**: Effect of MOX-F on MCF-7 cells at different concentrations

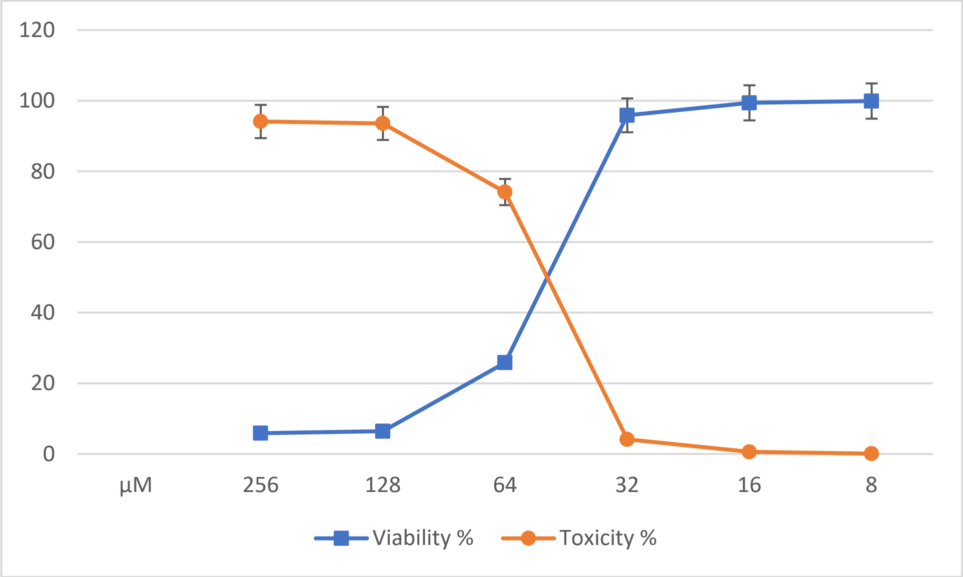

Figure **S44**: Effect of MOX-F on HepG 2 cells at different concentrations

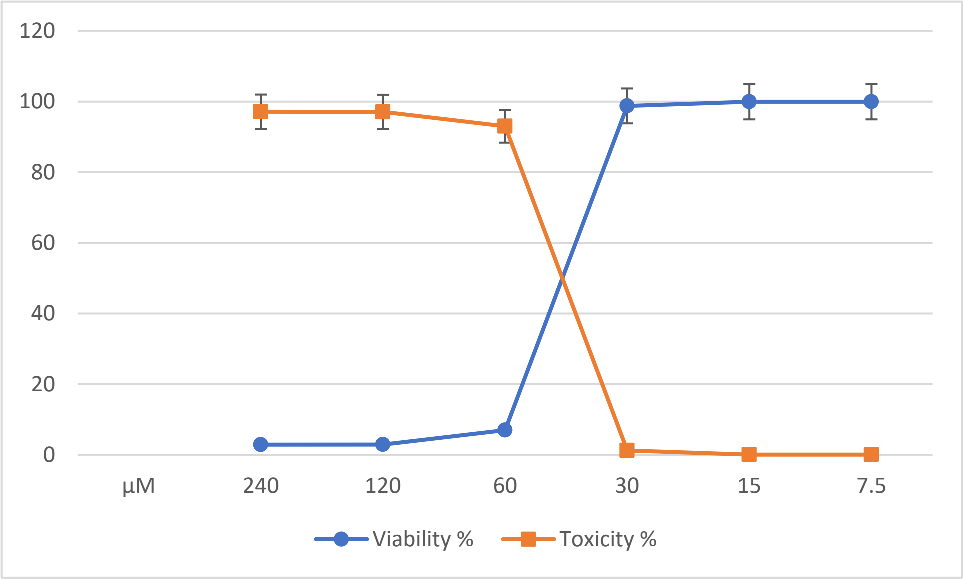

Figure **S45**: Effect of MOX-H on Vero cells at different concentrations

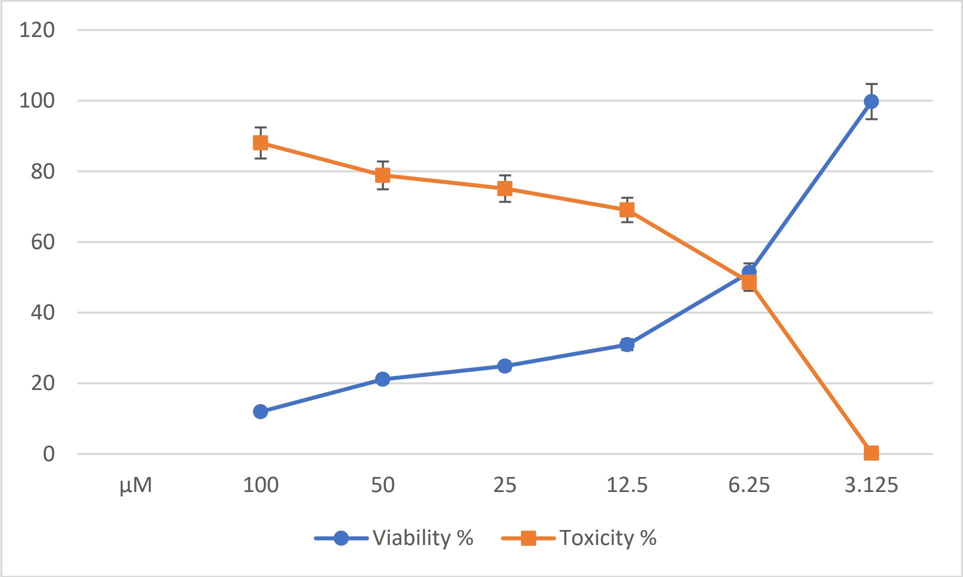

Figure **S46**: Effect of MOX-H on MCF-7 cells at different concentrations

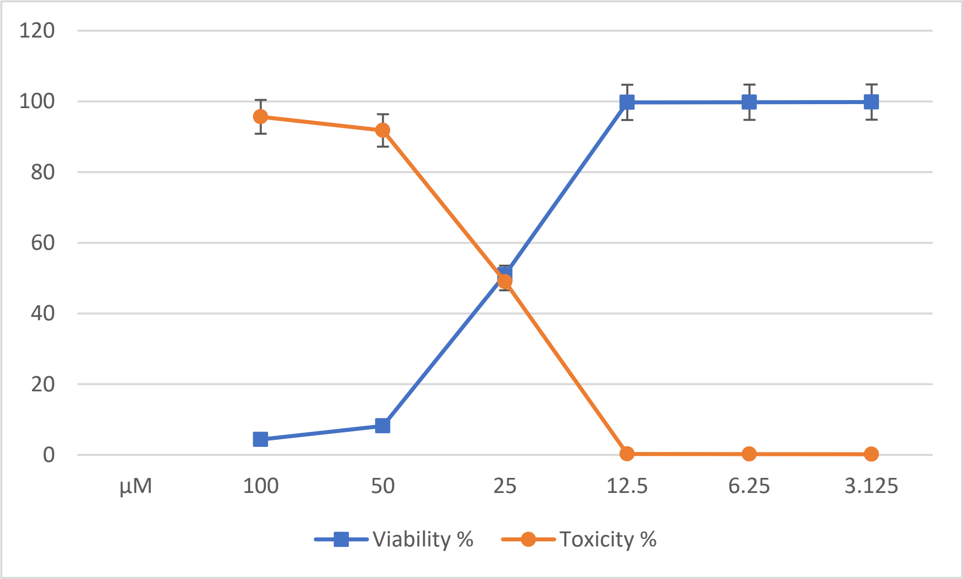

Figure **S47**: Effect of MOX-H on HepG 2 cells at different concentrations

Table S11. IC_{50} values for MOXF, MOX-H and MOXH-Cu (II) complex, against Vero (normal), MCF-7 (breast cancer), and HepG2 (liver cancer) cell lines.

Cell line	**Vero**	**MCF-7**		**HepG2**	
Compound	**IC_{50}± SD**	**IC_{50}± SD**	**SI**	**IC_{50}± SD**	**SI**
**MOX-F**	75.38± 0.021	69.50± 0.045	1.08	45.33± 0.187	1.66
**MOX-H**	42.69 ± 0.09	8.61 ± 0.07	4.96	30.37 ± 0.08	1.41
**Cu-MOXH**	32.54 ± 0.04	7.50± 0.07	**4.34**	16.96± 0.018	1.92

https://doi.org/10.3390/ijms27073057
